# Physiological attunement and flourishing: understanding the influence of relationships on health

**DOI:** 10.3389/fpsyt.2025.1614379

**Published:** 2025-08-01

**Authors:** Marco A. Vinhosa Bastos, Danilo Faria Braz, Ana Laura Manzan Porto, Khadija S. da Silva Cordeiro, Renata Boschi Portella, Douglas Alan Granger

**Affiliations:** ^1^ School of Medicine, Federal University of Mato Grosso do Sul, Campo Grande, Brazil; ^2^ Institute for Interdisciplinary Salivary Bioscience Research, University of California, Irvine, CA, United States

**Keywords:** adult, physiologic interdependence, cortisol, relationship, flourishment, wellbeing

## Abstract

**Background:**

Flourishing can be defined as the experience of life going well, a combination of feeling good and functioning effectively. High-quality relationships are essential to flourishing and long-term health. Physiological interdependence—such as synchronization of autonomic and endocrine systems—has been proposed as a mechanism supporting emotion regulation and social bonding.

**Methods:**

This scoping review maps the existing literature on physiological attunement in adult dyadic relationships. The review protocol was registered on the Open Science Framework (https://osf.io/295ge/) and followed JBI methodology for scoping reviews. PubMed and Scopus databases were searched. Eligible studies were original, quantitative, peer-reviewed articles published in English that examined physiological attunement in adult human dyads. Two reviewers independently screened and selected the studies.

**Results:**

A total of 62 studies were included. Attunement was observed in romantic partners, friends, strangers, and groups, involving heart rate, heart rate variability, skin conductance, respiration, cortisol, and alpha-amylase. Physiological attunement was shaped by relational context, emotional tone, individual traits (e.g., empathy, attachment style), and interaction features (e.g., touch, conflict, cooperation). While often linked to satisfaction, intimacy, and co-regulation, synchrony also appeared in distress contexts, sometimes reflecting stress contagion or co-dysregulation.

**Conclusion:**

Physiological attunement appears to be a context-sensitive process that may support or hinder wellbeing. It may represent a key biobehavioral pathway linking relationships to flourishing.

## 1 Introduction

The emerging concept of flourishing reflects a growing recognition of what constitutes holistic wellbeing (1). Its increasing use in wellbeing research can be traced back to the work of Keyes (2), who defined it as having “complete mental health … to be filled with positive emotion and to be functioning well psychologically and socially” (3). Another influential definition refers to it as “the experience of life going well … a combination of feeling good and functioning effectively” (4).

Although research aimed at assessing flourishing has expanded significantly over the past two decades, a universally accepted definition of the construct has yet to be established. The flourishing scales are generally subjective measurements of wellbeing. Rule et al. (3) compared seven scales that have been developed to measure flourishing among adolescent and adult populations. They found that the operationalization of wellbeing dimensions varies considerably between existing flourishing scales. Yet, they found that the measures share several commonalities as follows: multi-dimensionality, self-reporting of subjective wellbeing, an emphasis on everyday functioning, and relative stability across time.

According to VanderWeele (5), four key domains—family, work, school, and community—may serve as primary pathways through which flourishing is expressed across various areas of life, with their relative importance likely shifting across different stages of the lifespan. Flourishing is understood to encompass primarily psychological and social dimensions of human functioning (3).

Virtually every measure of health and wellbeing is improved by access to close social relationships and rich social networks (6). The *social baseline theory* states that close proximity to social resources is the baseline assumption of the human brain (6). This theory, which has bases on behavioral ecology and cognitive neuroscience, suggests that the human brain expects access to social relationships that mitigate risk and diminish the level of effort needed to meet a variety of goals (7). Social proximity would represent an innate, default or baseline strategy for human emotion regulation – one that does not require overt or deliberate action (6). Emotional regulation is considered a key skill for managing stress and to achieving flourishment (8).

Stress is traditionally conceptualized as a dynamic process triggered by actual or perceived environmental demands, which may be evaluated as either threatening or benign based on the individual’s available coping resources (9). The autonomic nervous system (ANS) and the hypothalamus-pituitary-adrenal axis (HPA axis) are considered the two main stress response systems of humans (9, 10). Objective measurement of physiological parameters that may vary according to the emotions experienced (like those linked to the ANS and to the HPA axis, e.g., cortisol measures) can lead to a better understanding of an individual`s psychological experiences.

A principal advantage of examining physiological responses during interpersonal interactions is the ability to explore theoretical questions that cannot be adequately addressed through self-reports or behavioral observations alone (11). Physiological data offer continuous insights into emotional states, including those that are unconscious or are not readily observable (12), and are not subject to the same demand effects that can bias self-reported data. Additionally, these responses can be monitored unobtrusively, enabling the assessment of psychological processes without disrupting the natural dynamics of interpersonal interactions (11). Shared physiological states are a phenomenon that has been investigated through these methods.

Physiological attunement refers to the temporal alignment of two or more people`s physiological states (13, 14). However, there is considerable diversity in the terminology used to refer to shared physiological states. Attunement, concordance, contagion, coregulation, coupling, covariation, entrainment, influence, linkage, and synchrony are some of the terms used to describe interdependence in partners` physiology. These terms sometimes reflect subtle conceptual distinctions regarding what is being assessed or denote specific analytical approaches. For instance, “coupling” and “synchrony” are often used to describe the correlation between partners’ physiological responses occurring at the same time point. In contrast, ‘‘attunement” and “coregulation” typically refer to the extent to which one partner’s physiological state predicts the other’s at a subsequent time point (15, 16). However, even these definitions are applied inconsistently across the literature.

The conceptual distinction between physiological synchrony and attunement—particularly as it pertains to concurrent versus lagged models—has gained attention in recent years, but it is not yet universally standardized or widely agreed upon. Many studies still use the term synchrony broadly and interchangeably with attunement, often without specifying whether the models employed are concurrent or lagged. The assumption that concurrent synchrony is purely situational and lagged synchrony inherently relational may oversimplify the dynamic and reciprocal nature of real-life interactions (17, 18). We argue that interpreting lagged physiological synchrony as definitive evidence of interpersonal, directional regulatory processes requires caution, as several confounding factors may influence the temporal structure of dyadic physiological data. For instance, individual differences in autonomic reactivity—such as variations in emotional responsiveness or health status—can yield time-lagged patterns that are intrapersonal rather than relational (19). Additionally, shared environmental stimuli or task characteristics may elicit temporally offset responses in both individuals (20). For these reasons, in the present work, we did not adopt a strict distinction between synchrony and attunement and used the terms interchangeably.

The widespread occurrence of synchrony in social interactions has become increasingly evident. This is largely due to advancements in continuous behavioral and physiological measurement techniques as well as the development of statistical methods capable of computing the concordance between two or more time series (14, 21, 22). Usually, to assess physiological attunement, the utilization of one or more of the following statistical approaches is necessary: correlation analysis, multilevel modeling, growth curve modeling, and cross-lagged panel analysis (23).

Autonomic mimicry enables emotion system coupling by allowing a receiver to implicitly mirror the physiological states of a sender—such as changes in pupil size, cardiovascular activity, and hormonal levels. Rooted in predictive coding mechanisms, this process helps the brain minimize prediction errors by aligning internal states with observed cues. Especially in infants, whose neocortex is underdeveloped, autonomic mimicry supports early emotion regulation by fostering adaptive responses and emotional attunement with caregivers (24).

Although autonomic mimicry is especially critical in infancy, it remains relevant in adults as a mechanism for emotion regulation. Adults continue to exhibit physiological mirroring, particularly in emotionally salient or intimate interactions. These responses reflect underlying predictive coding processes that help minimize emotional uncertainty and support co-regulation. While adults rely more on cognitive appraisal and learned regulation strategies, autonomic mimicry still facilitates emotional alignment and attunement, especially in contexts involving empathy, stress contagion, or social bonding (25–27).

Although the concepts of emotional contagion and physiologic attunement overlap in some ways, there are distinctions between them. Emotional contagion refers to the phenomenon where individuals unconsciously “catch” the emotions of others through automatic mimicry of facial expressions, vocalizations, postures, and movements, leading to shared emotional experiences. The process of emotional contagion appears to be facilitated by the mirror neuron system, which enables individuals to internally replicate observed behaviors and emotions (24).​ On the other hand, physiologic attunement involves the synchronization of physiological processes, such as heart rate, respiration, and hormonal levels, between individuals during social interactions, reflecting a state of mutual regulation and connection (28–30).

The literature presents a range of conceptual models for understanding mental health. One perspective suggests that mental health exists along a single continuum, extending from the presence of mental illness to states of happiness and wellbeing (3, 31). Within this framework, flourishing is positioned as the optimal end of the mental health spectrum, representing its most complete expression (3). Moreover, flourishing, conceived as a multidimensional and stable indicator of wellbeing, is inherently social in nature—an attribute rooted in the biological makeup of human beings (6). In its turn, physiological attunement appears to be an important mechanism for embodying the influence of sociality on organic functioning.

We propose that physiological attunement may represent a biological mechanism that supports, or in certain circumstances undermines, the experience of flourishing. Deepening the understanding of the relationship between social emotion regulation and flourishing may advance scientific insight into the prevention of emotional dysregulation— which is a barrier to wellbeing (32). Such insights may contribute to the development of more integrative therapeutic approaches, inform cultural and educational initiatives, and ultimately promote improved quality of life at both individual and societal levels.

Although there are some reviews on physiological attunement (11, 14) and at least one review on flourishing (3) available in the literature, to our knowledge, no existing review has synthesized current findings on physiological attunement in relation to the concept of flourishing. The present review addresses the research question concerning the occurrence of physiological synchrony across different types of adult relationships and subsequently examines how physiological attunement may influence individual wellbeing. We chose the scoping review approach because we intended to obtain a broad overview of the topic, identifying key concepts and mapping the literature, rather than testing a hypothesis (33).

## 2 Methods

### 2.1 Study design

A scoping review was carried out following the Arksey and O’Malley (34) guidelines: 1) identifying the research question, 2) identifying relevant studies, 3) study selection, 4) charting the data, and 5) collating, summarizing, and reporting results. The review also followed the JBI methodology for scoping reviews. The review protocol was registered on the Open Science Framework (OSF) (https://osf.io/295ge/). Institutional ethics board approval was not required.

### 2.2 Databases and search strategy

The scientific databases (SCOPUS and PubMed) were consulted, using keywords from the Medical Subject Headings (MeSH). The search strategy was [(physiologic interdependence OR physiologic attunement OR physiologic synchronization OR physiologic synchrony OR physiologic coregulation OR emotional contagion] AND [dyad* OR couple OR spouse OR partner OR romantic OR friend) AND (adult)]. To avoid missing important papers, we repeated the search using the keyword ‘cortisol’, as follows: [(cortisol interdependence OR cortisol attunement OR cortisol synchronization OR cortisol synchrony OR cortisol coregulation OR emotional contagion] AND [dyad* OR couple OR spouse OR partner OR romantic OR friend) AND (adult)]. Cortisol was selected as a focal point in the search strategy due to the well-established role of the hypothalamic–pituitary–adrenal (HPA) axis in responding to emotions and stress (35), as well as its widespread use as a biomarker in biological psychology and psychiatry. Two researchers performed the searches following the same strategy. The search took place between February 1^st^ and March 30^th^ 2025. Google Scholar was consulted for additional publications and grey literature, in addition, reference lists of included papers was verified.

### 2.3 Inclusion and exclusion criteria

This scoping review included quantitative studies published in peer-reviewed journals, in English, that investigated physiological attunement occurring in adult dyadic relationships. We limited the search to studies published since 2000 to prioritize research that employs contemporary statistical approaches and advances in technology for assessing physiological synchrony. This time frame reflects the growing availability of continuous physiological monitoring tools and the development of dynamic dyadic analysis methods that have enhanced the precision and ecological validity of synchrony research (15, 22). Review articles, editorials, letters to the editors, or case reports, were excluded from this review. Studies addressing physiological attunement in the context of mother-infant and father-infant relationships were also excluded. We decided to also exclude studies on emotional contagion because, as pointed out in the Introduction, this concept differs from physiological attunement. This review aimed to investigate a normal interpersonal physiological phenomenon (attunement), so studies involving specific diseases were also excluded. Studies assessing therapist–patient interactions were excluded because their structured, asymmetrical nature differs from every day, reciprocal relationships, potentially limiting the review’s focus on naturalistic interpersonal flourishing.

### 2.4 Data extraction

Five researchers were responsible for extracting the data independently. In the first stage, duplicate records were eliminated. Then, in the second stage, the studies were independently selected and analyzed by two researchers by reviewing titles and abstracts. In the third stage, the selected studies were analyzed by reading the full text to verify that they continued to meet the inclusion criteria.

In case of records with no complete agreement, a third researcher assessed them. The results of each stage were agreed upon, and discrepancies were resolved with the arbitration of a third researcher and, if necessary, a fourth researcher.

Finally, the following data was extracted: authors, year, country, purpose of the study, study design, sample characteristics, and major findings. The researchers used Microsoft Windows Office for both screening and citation management, without employing any specialized software for these tasks.

### 2.5 Generative artificial intelligence disclosure

Chat PDF AI was employed for data extraction from the included articles and Open AI Chat GPT-4.0 was used as a starting point in the writing of the Results and Discussion sections. Data extraction was supervised. The authors carefully reviewed and edited the AI generated texts as needed and take full responsibility for the content of the manuscript.

As this is a scoping review rather than a systematic review, we opted not to perform a formal assessment of study quality or risk of bias (33).

## 3 Results

**Figure 1:**
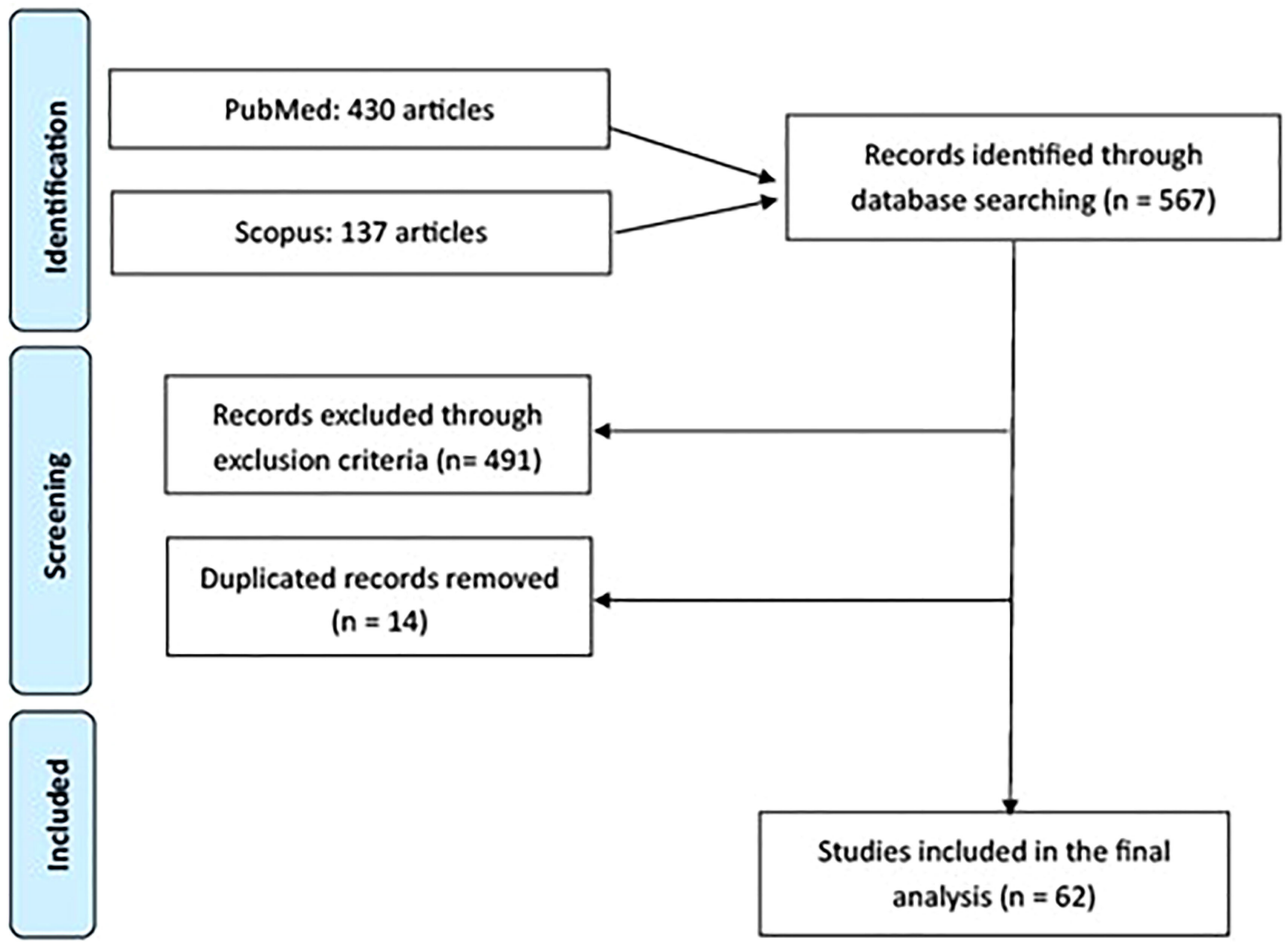
Flow chart of study screening according to PRISMA-ScR (Preferred Reporting Items for Systematic Reviews and Meta-Analyses - extension for scoping reviews) guidelines (33).

The stage 1 (identification) of the search led to the retrieval of a total of 567 articles (**Figure 1**).

In stage 2 (screening and eligibility), the application of the inclusion and exclusion criteria, plus removal of duplicated articles, resulted in the exclusion of 503 articles and the inclusion of 62 articles in final analysis.

The most common reasons for exclusion of articles were: studies focusing on parent–infant relationships (mother–infant or father–infant); studies involving samples composed exclusively of children or adolescents; theoretical, review, and methodological articles; studies centered on specific diseases; and studies focused on emotional contagion.

In stage 3 (critical analysis of studies), all 62 studies found were evaluated in terms of purpose, study design, sample characteristics, and main findings. The screening of references lists of initially included articles failed to add any extra publication for final analysis. **Figure 1** illustrates the results of the study screening strategy.

This scoping review synthesized evidence from 62 quantitative studies examining physiological attunement in adult interpersonal interactions. Overall, findings support the existence of physiological synchronization across multiple systems—particularly the autonomic nervous system (ANS) and hypothalamic-pituitary-adrenal (HPA) axis—in a variety of dyadic and group contexts. These studies were charted into five analytical categories based on the physiological system investigated and the context of interpersonal interaction: (1) autonomic nervous system attunement in romantic partners during conflict situations; (2) autonomic attunement in romantic partners during non-conflict interactions; (3) cortisol attunement in romantic partners; (4) autonomic attunement in non-romantic dyads (friends, strangers, and groups); and (5) cortisol attunement in non-romantic dyads. All studies involved adult participants and employed quantitative methodologies.

The five analytical categories were derived inductively from the reviewed literature to facilitate a more coherent organization of the data. The present Results section brings 5 tables summarizing the studies that were included in each of the abovementioned analytical category. It also includes brief observations on sample characteristics, patterns and trends in methodology, as well as what the reviewed studies collectively show about each form of attunement.

### 3.1 Autonomic nervous system attunement in romantic partners during conflict situations

Twelve studies examined autonomic nervous system (ANS) attunement in romantic couples during conflictual or emotionally charged interactions. **Table 1** briefly describes the 12 included studies. Measures included heart rate (HR), heart rate variability (HRV), respiratory sinus arrhythmia (RSA), electrodermal activity (EDA), and blood pressure. The majority of studies employed laboratory-based paradigms (e.g., conflict discussions, emotionally salient conversations, structured relational tasks), though a few incorporated longitudinal or semi-naturalistic designs. Most samples comprised heterosexual couples, with average ages ranging from early 20s to mid-50s, and relationship durations spanning from newly formed to long-term marriages. Most studies employed used statistical techniques such as multilevel modeling, actor-partner interdependence modeling, and correlational analysis. Geographically, studies were predominantly conducted in North America and Western Europe, with limited cultural or demographic diversity.

**Table 1:** Details of the studies examining physiological attunement of autonomic nervous system parameters in romantic partners, during conflict situations.

Authors, year and country	Purposes of the study	Study design and statistical analysis	Sample characteristics	Major findings
Reed et al., 2013 (USA) (36)	To explore whether partner influence predicts patterns of physiological linkage during health-related conversations among romantic partners, assessing how different perceptions of influence (both positive and negative) affect physiological responses.	Participants took part in a laboratory session where physiological measures were taken during a conversation between romantic partners about their influence on each other’s health behaviors. The physiological parameters assessed were: blood pressure, inter-beat interval, and skin conductance. Researchers categorized the levels of partner influence as “high” or “low”, according to the partners’ perceptions and behaviors related to how they attempt to influence each other’s health-related behaviors. Statistics: PROC MIXED procedure in ‘Statistical Analysis System’ software.	Total sample (N) = 88 (44 heterosexual romantic couples), average age 31.7± 12.7 years.	Physiological linkage of blood pressure varied depending on perceived partner influence. At low levels of influence, physiological linkage was characterized as “anti-phase” (i.e., partners’ physiological responses moved in opposite directions). At high levels of partner influence, the physiological responses were “in-phase” (i.e., reciprocal changes in responses).
Nelson et al., 2016 (USA) (30)	To investigate the effects of dispositional and experimentally induced perspective-taking (PT) on physiological attunement between romantic partners during a conflict resolution task.	Laboratory-based experimental dyadic design. Couples rated their trait perspective-taking one week before a conflict resolution session. Participants were assigned to one of three conditions immediately before the conflict task: perspective-taking; mindfulness; and control condition (focused on their own perspective). Saliva samples were collected to measure alpha-amylase to assess autonomic nervous system activity during the conflict resolution task. Statistics: multilevel modeling.	Total sample (N) = 206 (103 heterosexual romantic couples), average age 21.3± 6.1 years.	Couples in the perspective-taking condition displayed greater autonomic attunement compared to those in the mindfulness and control conditions. Female partners’ dispositional perspective-taking enhanced the efficacy of the perspective-taking induction on couples’ attunement. Negative conflict behaviors were associated with decreased autonomic attunement. Autonomic attunement was linked to the emotional outcomes of the conflict discussion (i.e., less attunement predicted increased post-conflict negative affect in female partners).
Wilson et al., 2018 (USA) (37)	To investigate the associations between couples’ heart rate variability (HRV) synchrony during conflicts and subsequent inflammatory responses throughout the day.	Each couple participated in two study visits (spaced 4.4 weeks apart, on average). Couples engaged in a marital problem discussion involving contentious topics identified in prior inventories. Heart rate variability was measured continuously throughout the visit. Blood samples were collected at multiple times and were analyzed to quantify inflammatory markers. Statistics: Linear mixed models.	Total sample (N) = 86 (43 married couples), average age 38 ± 8.2 years, average duration of marriage was 11.5 years.	Stronger HRV synchrony during conflict was associated with higher levels of three inflammatory markers (IL-6, stimulated TNF-α, and sVCAM-1) across the day (independently of marital quality and other demographic factors). Couples exhibiting closely tracked HRV changes during conflicts were more likely to demonstrate greater negative affect reactivity.
Caldwell et al., 2019 (Canada) (38)	To examine whether respiratory sinus arrhythmia (RSA), a physiological marker related to self-regulation, moderates the impact of rumination, a maladaptive emotion regulation strategy, on couples’ conflict.	Dyadic longitudinal design, focusing on the interactions and dynamics between two individuals in a romantic relationship. Measured rumination and RSA at baseline and assessed couples’ conflict at three different time points over 12 months. Statistics: Actor-partner interdependence modeling.	Total sample (N) = 168 (84 cohabiting, heterosexual couples raising young children), average age 34.60 ± 4.70 years, average duration of relationship 9 ± 4.32 years.	Rumination from both partners was independently associated with increased couples’ conflict over the 12 months. High RSA in an actor (partner) was found to attenuate the positive association between the partner’s rumination and the couples’ conflict. Couple`s conflict levels remained stable on average across the year.
Coutinho et al., 2019 (Portugal) (39)	To explore the presence of physiological synchrony during interactions between romantic partners and compare synchrony levels during positive versus negative interactions.	Each couple was positioned on separate sofas to allow them to view and interact with each other in a comfortable setting. Then, couples engaged in a structured interaction task that included discussing various topics. The interactions were designed to elicit varying emotional valences (positive and negative). Physiological synchrony was measured by analyzing the real-time patterns of electrodermal activity between partners during these discussions. Statistics: Hierarchical Regression Analysis, Multiple Linear Regression Analysis.	Total sample (N) = 64 (32 heterosexual romantic couples), average age 32.3 ± 10.2 years. Average duration of romantic relationships was 9.4 ± 8.0 years.	Significant electrodermal activity synchrony was observed during interactions, with synchrony being higher in negative interactions compared to positive ones. Physiological synchrony during negative interactions was elevated, while during positive interactions, higher synchrony was associated with males scoring higher in dyadic empathy.
Smith et al., 2020 (USA) (40)	To investigate the association between behavioral measures of marital conflict (both expression and exposure to negativity) and cardiovascular reactivity (CVR), particularly blood pressure changes, during marital conflict discussions. It also aimed to differentiate the effects of the individual’s own conflict behaviors from those of their partner.	Each couple participated in a structured laboratory task where they discussed a selected topic of disagreement (for 10 minutes). Blood pressure reactivity was quantified. Structural Analysis of Social Behavior (SASB) coding system was used to evaluate the conflict behaviors displayed by partners. Statistics: Actor-partner interdependence modeling.	Total sample (N) = 292 (146 heterosexual middle-aged couples), average age males 45.8 years (range 32–54), females 43.9 (range 32–54), average duration of romantic relationships 18.4 years.	Only the individual’s own expressions of negative behavior (reflected in hostility and dominance) were significantly associated with increases in both systolic blood pressure (SBP) and diastolic blood pressure (DBP). The behaviors displayed by partners did not significantly predict changes in blood pressure.
Chen et al., 2021 (USA) (41)	To explore how physiological linkage relates to shared emotional experiences (both positive and negative) in couples and to determine how these linkages reflect relational functioning.	Each couple had a 15-minute conflict conversation in a laboratory setting. It utilized behavioral coding, categorizing each second of conversation into four emotion categories: shared positive emotion, shared negative emotion, shared neutral emotion, or unshared emotion. They measured heart rate, skin conductance, and finger pulse amplitude. Statistics: Correlational analyses (Pearson’s correlations) and nonparametric statistical tests (Mann-Whitney U tests).	Total sample (N)=312 (156 opposite-sex long term married couples), average age 54.1 ± 0.8 years (males) and 52.8 ± 0.8 years (females), married for at least 15 years.	Shared positive emotions were significantly associated with greater in-phase physiological linkage compared to negative and neutral emotions. There were higher quality interactions and marital satisfaction during shared positive emotions, supporting a connection between physiological synchronization and relational quality.
Coutinho et al., 2021 (Portugal) (42)	To explore the presence of cardiac synchrony in romantic partners during positive and negative interactions and to assess the association of synchrony with self-report measures of empathy and relationship satisfaction.	Couples underwent a structured interaction task in a lab setting, discussing both positive and negative aspects of their relationship. Heart rate (HR) and heart rate variability (HRV) were recorded during the interaction. Pre-task self-report measures were also taken, including empathy (Interpersonal Reactivity Index) and relationship satisfaction (Revised Dyadic Adjustment Scale). Statistics: Multilevel modeling	Total sample (N) = 54 (27 heterosexual romantic couples), average age 32.4 ± 7.5 years (males) and 33.6 ± 7.8 years (females).	There was cardiac synchrony in couples during discussions, with negative (antiphase) synchrony of HRV and positive (in-phase) synchrony of HR. When partners’ heart rates synchronized positively, they tended to report higher levels of empathic concern.
Margolin et al., 2022 (USA, Canada & Israel) (43)	To explore the relational, emotional, and physiological impacts when couples discuss personal losses.	Structured laboratory session that included repeated 10-minute discussions about desired changes in their relationships or about personal losses. Participants were monitored for physiological arousal via skin conductance responses (SCR). Following each discussion, participants filled out a post-discussion questionnaire that assessed their emotional experiences. Statistics: Actor-Partner Interdependence Model.	Total sample (N) = 228 (114 heterosexual romantic couples), average age 22.5 ± 2.4 years	Narrating personal loss elicited more vulnerable emotions (e.g., sadness). Both narrating and listening to loss significantly increased self-reported closeness between partners compared to discussions on desired relationship changes. Women showed decreasing skin conductance responses when narrating their losses, indicating lower physiological arousal. In contrast, both male and female listeners exhibited increasing SCRs while listening to their partner’s narration, reflecting the emotional load of being a responsive partner.
Barden et al., 2025 (USA) (44)	To investigate how posttraumatic stress symptoms (PTSS) affect self-regulation and coregulation of respiratory sinus arrhythmia (RSA) in couples following an acute stress induction.	After baseline tasks (viewing neutral video together then discussing about the video), one partner (randomly selected as the stressed partner) was assigned to watch a stress-inducing film clip, which depicted a scene of sexual violence. After that, the stressed partner reunited with their partner (the nonstressed partner) and they then participated in a postfilm dyadic discussion. RSA was monitored during both the baseline and postfilm discussion tasks. Statistics: Repeated measures actor-partner interdependence model.	Total sample (N) = 86 (43 romantic couples; 71% heterosexual), average age 28.5 ± 10.7 years (males) and 27.0 ± 9.0 years (females). All participants had a history of trauma.	Both partners demonstrated self-regulation of RSA during the study. The nonstressed partner showed enhanced self-regulatory efforts during the dyadic discussion after the stressor task. Greater PTSS in the nonstressed partner was associated with a weakening of both self-regulation (RSA changes in oneself) and coregulation (RSA changes influenced by one partner’s state affecting the other).
Kykyri et al., 2025 (Finland) (45)	To investigate the association between physiological synchrony (both in movements and electrodermal activity) and the emotional aspects along with the conversational structure within one emotionally intense couple therapy session.	An exploratory single case study design was employed. The method involved detailed qualitative analysis of a couple therapy session along with quantitative analysis of physiological synchrony. Video recordings were utilized to capture interactions during the therapy session, where participants’ electrodermal activity was also recorded to assess physiological synchrony. The session was divided into topical episodes for analysis. Statistics: Multiple linear regression analysis.	The study analyzed a couple therapy session involving two clients (a couple) and two therapists, allowing for interaction dynamics (six different dyadic interactions).	Physiological synchrony, measured through electrodermal activity (EDA) and movements, was significantly influenced by emotional expressions during the therapy session. EDA synchrony was strongly associated with emotional intensity. Higher physiological synchrony was observed during conversations directly addressing the couple’s relationship issues.
Shimshock et al., 2025 (USA) (46)	To investigate the variability in physiological synchrony among romantic couples during emotionally charged conversations regarding their future, focusing on how partner behaviors influenced synchrony.	A dyadic design, was employed. Data were collected during emotionally salient discussions focusing on future relationship implications following partners disclosing positive personal news (e.g., job offers). Each partner played distinct roles: one as the “discloser” and the other as the “responder.” Cardiovascular measures (cardiac interbeat intervals) were used during discussions. Statistics: ANOVA.	Total sample (N) = 158 (79 romantic couples, 96% heterosexual, average age 20.1± 1.3 years.	Physiological synchrony varied significantly based on the behaviors exhibited during discussions. Higher synchrony was observed when:disclosers spoke less (more talk time from disclosers led to less physiological covariation); responders displayed less withdrawal and neglect; both partners exhibited greater positive emotionality.

Conflict discussions consistently elicited in-phase or anti-phase physiological synchrony, modulated by factors such as partner empathy (39, 42), perspective-taking (30), or trauma history (44). Several studies linked higher attunement to emotional or relational outcomes, including increased marital satisfaction (41), while others identified potential physiological costs, such as elevated inflammatory markers following high HRV synchrony during conflict (37).

### 3.2 Autonomic nervous system attunement in romantic partners during non-conflict situations

Nine studies investigated autonomic attunement in romantic partners during affiliative, cooperative, or intimate interactions. **Table 2** briefly describes the 9 included studies. The studies applied tasks involving eye-gazing, interpersonal touch, emotional sharing, imitation, and sexual activity. Measured parameters included HR, HRV, respiration, and skin conductance. Most studies used multilevel statistical approaches such as multilevel modeling or oscillator-based models. Samples consisted mostly of heterosexual couples in young to early adulthood, with average ages ranging from approximately 23 to 30 years, and relationship durations varying from a few months to several years. All studies were conducted in North America or Europe, and only one included same-sex dyads.

**Table 2:** Details of the studies examining physiological attunement of autonomic nervous system parameters in romantic partners, during non-conflict situations.

Authors, year and country	Purpose of the study	Study design and statistical analysis	Sample characteristics	Major findings
Helm et al., 2012 (USA) (15)	To investigate physiological synchrony between romantic partners by examining associations in their heart rate and respiration patterns during different interaction tasks.	A dyadic experimental design was used, with physiological responses of both partners in a romantic relationship being recorded during three tasks: baseline (resting), gazing into each other’s eyes, and an imitation task where they attempted to synchronize their physiological responses. Statistics: Damped Linear Oscillator (DLO) Models.	Total sample (N)=64 (32 heterosexual couples), average age 30.3 ± 11.9 years, average relationship duration 8.2 ± 8.6 years (range 1.5-34.8)	Couples showed significant physiological synchrony in both heart rate and respiration, especially during the gazing (greater synchrony) and imitation tasks. Relationship satisfaction and attachment styles influenced synchrony. Couples with higher relationship satisfaction exhibited dampened synchrony. Avoidant individuals showing weaker synchrony during resting and gazing but stronger synchrony in the imitation task.
Chatel-Goldman et al., 2014 (France) (47)	To investigate how interpersonal touch influences physiological (autonomic) coupling between romantic partners and to explore the potential role of touch in promoting empathy and emotional connection during interactions.	Ecological paradigm where romantic partners interact only via touch. Empathic states were manipulated. There were two main conditions: ‘No Touch Condition’: For the first five runs, and ‘Touch Condition’: In the final five runs (participants allowed to touch each other’s hands and forearms). Simultaneously, autonomic activity (skin conductance, pulse, respiration) was assessed. Statistics: Repeated-measures ANOVA.	Total sample (N)=28 (14 heterosexual romantic couples), mean age for women 25.4 ± 3.5 years, and for men 26.1 ± 3.7 years. Average relationship duration 2.9 years (range 6 months to 5 years).	Interpersonal touch significantly increased the coupling of electrodermal activity between the partners. Participants reported feeling more intense emotions and perceived a greater sense of coupling when in the “Touch” condition compared to the “No Touch” condition. The effect of touch on physiological coupling did not depend on the intensity or valence of the emotions experienced by the participants.
Karvonen et al., 2016 (Finland) (48)	To investigate whether there is a significant sympathetic nervous system synchronization (assessed via electrodermal activity) between couples, therapists, and client-therapist dyads during therapy.	The study adopted a multifactor design, focusing on the interactions between clients and the therapists as well as among co-therapists, during couple psychotherapy sessions. Electrodermal activity, heart rate and respiratory pattern of participants were measured during the sessions. Statistics: Correlations analyses, one-way ANOVA.	Total sample (N)=40 Consisting of 10 couples (20 clients) and 10 therapists dyads (20 therapists working in pairs, the majority [80%] in male-female pairs). Average age: Clients= 40.1 ± 9.6 and Therapists= 41.1 ± 6.7 years. Mean relationship duration for the couples: 15.7 ± 8.9 years. Reasons for seeking therapy: relationship issues, external life stressors, past violence, or current relationship violence.	Sympathetic synchrony was observed in 85% of all dyads during therapy sessions. Unexpectedly, therapist pairs showed the highest levels of synchrony, followed by client-therapist dyads, while couples exhibited the lowest synchrony. Authors suggest that couples’ lower synchrony might be due to relationship difficulties or emotional disconnection.
Goldstein et al., 2017 (Israel) (49)	To investigate how interpersonal touch influences physiological coupling (heart rate and respiration synchronization) in romantic partners when experiencing shared empathy for pain.	The experiment included six counterbalanced conditions: no pain-alone, pain-alone, partner touch-no pain, partner no touch-no pain, partner touch-pain, and partner no touch-pain. Physiological responses (heart rate and respiration rates) were measured, examining the cross-partner dynamics. Trait empathy was measured through Interpersonal Reactivity Index (IRI) instrument. Statistics: Continuous Linear Oscillator (CLO) model.	Total sample (N)=44 (22 heterosexual romantic couples), mean age for men was 26.4 ± 2.3 years, and for women, 25.6 ± 1.9 years.	Touch significantly increased physiological synchronization (heart rate variability and respiration patterns) during the pain condition. Dyads with high empathic partners showed notably greater synchronization.
Vanutelli et al., 2017 (Italy) (50)	To investigate autonomic synchronization in dyads during the construction of a social bond, artificially induced by cooperative interaction and subsequent social feedback.	Participants completed an attentional couple game that required them to synchronize their responses. Feedback was integral to the task, designed to enhance the sense of cooperation and social bonding among participants. Autonomic parameters (heart rate and skin conductance levels) were measured over the course of the task. Statistics: Repeated measures ANOVA and correlational analyses.	Total sample (N)=24, average age 22.9 ± 1.2 years, 42% female. Each participant was paired into 12 dyads of the same sex, matched for age. Individuals in each dyad were not familiar with each other before the experimental session.	Higher heart rates were noted in the first part of the task, likely due to increased cognitive demand and the social dynamics involved. After the provision of social feedback, there was a significant increase in autonomic synchrony, particularly in electrodermal activity. Skin conductance levels showed exponential increases in synchronization during the second half of the task.
Freihart & Meston, 2019 (USA) (51)	To investigate the relationship between physiological synchrony (PS) and sexual satisfaction among heterosexual couples.	Couples participated in a series of laboratory tasks involving physiological measurements. Couples completed survey measures and were connected to an electrocardiogram, while engaging in baseline, gazing, and mirroring tasks. Statistics: Multilevel modeling.	Total sample (N)=56 (28 heterosexual romantic couples), mean age 25.9 ± 12.3 years.	Physiological synchrony was detected, with males reliably predicting the heart rate of their female partners and vice versa. A significant interaction effect was observed between sexual satisfaction and synchrony, during the mirroring task. The results suggested that couples who were more sexually satisfied manifested greater physiological synchrony.
Schacter et al., 2020 (USA) (52)	To investigate whether young couples’ feelings of closeness to and annoyance with each other during the day are associated with their overnight cardiovascular activity, specifically heart rate.	Participants completed hourly surveys reporting feelings of closeness and annoyance towards their partners, and their overnight heart rate was measured using wearable electrocardiogram biosensors. Statistics: Actor-partner interdependence models.	Total sample (N)=126 (63 heterosexual couples), mean age 23.1 ± 3.0 years.	No significant actor effects were found; individual participants’ own feelings of closeness or annoyance during the day did not affect their overnight heart rate. Women’s feelings of closeness predicted lower overnight heart rates in male partners when women felt closer during the day. Conversely, higher levels of women’s annoyance predicted elevated overnight heart rates in men, when closeness was low.
Qaiser et al., 2023 (Canada & USA) (53)	To investigate whether physiological synchrony emerges during empathy-inducing activities and to explore the relationships between trait and state measures of cognitive and affective empathy with physiological synchrony during the activity.	The couples participated in an empathy-inducing activity consisting of sharing a previous experience of suffering while their partner listened and responded. The physiological responses (electrocardiography, skin conductance levels, and respiratory sinus arrhythmia) of the couples were monitored throughout the process. Statistics: Multilevel modeling.	Total sample (N)=222 (111 romantic couples, predominantly heterosexual), average age 26.8± 7.2 years.	A physiological synchrony was noted in skin conductance reactivity and interbeat interval reactivity, though only when the disclosing partner was female. No synchrony was observed in respiratory sinus arrhythmia reactivity. Physiological synchrony did not correlate with other established measures of empathy.
Freihart & Meston, 2024 (USA) (16)	To examine how physiological synchrony (PS) develops during sexual activity and how it differs from non-sexual interactions within established couples.	Dyadic psychophysiological approach, focusing on the analysis of interactions between both partners during sexual and non-sexual activities. The study occurred in a naturalistic environment, specifically at participants’ homes. Heart rate and heart rate variability (HRV) were measured while participants engaged in sexual activity and a baseline task, to compare levels of synchrony. Specific sexual conditions included prescribed positions and freeform interactions. Statistics: Linear regression model.	Total sample (N)=116 (58 heterosexual romantic couples), mean age 23.3 ± 5.3 years, average relationship length 3.5 years.	The magnitude of PS was notably higher during sexual encounters compared to non-sexual interactions. Synchrony developed in specific sexual contexts, particularly during foreplay and freeform conditions, where partners could respond dynamically to each other. In contrast, PS was not achieved in a prescribed position condition, indicating the importance of natural and preferred sexual behaviors in facilitating synchrony.

Overall, physiological attunement was consistently observed across conditions involving emotional connection or physical intimacy. For instance, interpersonal touch reliably enhanced autonomic coupling (47, 49), and greater attunement was associated with higher sexual satisfaction (51) and stronger empathic connections (53). Conversely, constraints on natural interaction—such as prescribed sexual positions—diminished synchrony (16). In some cases, synchrony was asymmetrical or context-dependent, such as when only female partners’ emotional states predicted autonomic reactivity in male partners (52, 53).

### 3.3 Cortisol attunement in romantic partners

Twenty studies examined hypothalamic-pituitary-adrenal (HPA) axis attunement via salivary cortisol levels in romantic couples. **Table 3** briefly describes the 20 included studies. These studies employed both laboratory paradigms (e.g., conflict discussions, Trier Social Stress Test) and ecological momentary assessments (e.g., daily diary or multiday cortisol sampling). The studies investigated both younger and older adult couples across diverse countries, including the USA, Germany, Portugal, Israel, China, and Canada. Sample sizes varied substantially, ranging from 56 to over 600 participants, with most studies focusing on heterosexual couples and a few including older populations (e.g., 68, 70). The statistical methodologies employed were robust and varied, including dyadic multilevel modeling, actor-partner interdependence models, longitudinal growth curve modeling, and cross-lagged regressions. The conceptual heterogeneity in how “cortisol attunement” was defined and interpreted across studies is noteworthy.

**Table 3:** Details of the studies examining physiological attunement of cortisol in romantic partners.

Authors, year and country	Purpose of the study	Study design and statistical analysis	Sample characteristics	Major findings
Heffner et al., 2006 (16)	To examine how different communication patterns (specifically wife demand/husband withdraw) during marital conflict relate to the cortisol responses of older spouses, and to explore the impact of perceptions about these communication patterns on physiological responses.	Couples engaged in discussions designed to elicit conflict, during which physiological responses (salivary cortisol levels) were measured. Behavior was observed and self-reported perceptions of communication patterns within marital conflicts were assessed. Statistics: Regression analyses.	Total sample (N)=62, (31 heterosexual older couples), mean age 66.7 ± 5.0 years (range: 55–77), average length of marriage: 42.3 ± 1.7 years.	Higher levels of wife demand/husband withdraw communication patterns were significantly associated with increased cortisol responses in wives during conflict discussions. Perceived patterns of communication (self-reported by the spouses) were more strongly correlated with cortisol responses than the actual behaviors observed during the conflict. Wives showed a more pronounced physiological response (increased cortisol levels) to the conflict discussion than husbands.
Klumb et al., 2006 (Germany) (54)	To examine the physiological costs and benefits (as indicated by cortisol concentration) related to the time each spouse spent on productive activities, which included both formal (market work) and informal (household work) roles.	Participants maintained diary reports on their activities over six sampling days, documenting how much time they allocated to various tasks, including: market work (paid employment), household work, childcare, leisure activities, personal activities. Saliva samples for cortisol assaying were obtained after completing their activity reports. Statistics: Hierarchical linear models and multilevel modeling. Actor-partner interdependence models.	Total sample (N)=104, 52 heterosexual dual-earner couples, mean age 37 ± 4.9 years, each couple had at least one child under the age of 5.	Results demonstrated that couples were mutually influenced by each other’s work-related activities. High levels of marketwork (paid employment) led to increased cortisol levels in both partners, while supportive household work from one spouse had the potential to alleviate stress for the other (cortisol levels decreased).
Laurent & Powers, 2007 (USA) (55)	To investigate how temperament and attachment styles predict hypothalamic-pituitary-adrenal (HPA) axis response during conflict discussions in adult couples.	The study utilized a longitudinal design as part of a larger research project on factors contributing to depression. Participants attended to a laboratory setting where they engaged in discussions about unresolved conflicts while physiological data (salivary cortisol samples) were collected to assess HPA reactivity.	Total sample (N)=398, (199 heterosexual couples), mean age: male partners: 19.1 ± 0.8 years, female partners: 19.4 ± 08 years.	Higher attachment anxiety in partners was associated with increased cortisol response during conflict discussions. Attachment avoidance showed more complex effects. Female partners’ cortisol responses were significantly affected by the emotionality of their male partners, particularly when the males were high in emotionality and low in attachment avoidance.
Saxbe & Repetti, 2010 (USA) (28)	To explore the associations between couples’ cortisol levels and mood states, investigating how these physiological and emotional states are interlinked and whether marital satisfaction moderates these associations.	Longitudinal observational design, collecting data on multiple occasions across three days to examine the associations between partners’ cortisol levels and mood states. The assessment of mood states was conducted through self-report measures collected simultaneously with the cortisol saliva samples. Salivary cortisol was measured at multiple time points. Statistics: Dyadic multilevel modelling	Total sample (N)=60, 50% female, average age 41 years, divided in 30 opposite-sex married couples.	Husbands’ and wives’ cortisol levels were positively associated, suggesting that when one partner has a higher-than-usual cortisol level, the other partner tends to have a similar response. This association was stronger among those with lower marital satisfaction.
Liu et al., 2013 (USA) (56)	To investigate the daily dynamics of cortisol synchronization between spouses and to explore how this synchronization is influenced by specific characteristics of the marital relationship.	Couples were interviewed over eight consecutive days about daily experiences. From Day 2 to Day 5, saliva samples were collected for cortisol measurement to assess diurnal patterns (cortisol awakening response – CAR - and diurnal cortisol slope). Variables related to marital quality were also measured: spousal support and spousal strain (level of stress and disagreement experienced within the relationship). Statistics: Multilevel modeling. Cross-lagged regression analysis.	Total sample (N)=56, (28 heterosexual couples, married or cohabiting), mean age 40.2 ± 9.2 years, average length of the relationships 12 ± 11.7 years.	Husbands and wives showed synchronized diurnal cortisol patterns over days. When one partner’s cortisol levels were higher or lower than their personal average, the other partner’s levels also tended to adjust accordingly. Lagged relationships were seen: an individual’s cortisol level on a specific day was systematically influenced by spouse’s cortisol from the previous day. Higher levels of marital strain and disagreement were associated with stronger synchrony in cortisol patterns (CAR). Higher perceived spousal support was associated with stronger stability in the CAR for both partners.
Papp et al., 2013 (USA) (57)	To examine the associations between cortisol levels of husbands and wives, explore the physiological synchrony in their cortisol levels, and investigate the effect of emotional and physical connectedness on these associations.	Salivary cortisol levels were collected from couples at several (7) time points during the day across two typical weekdays. Emotional connectedness (measured via feelings of loneliness and being together) and physical presence (time spent together versus alone) were also assessed. Statistics: Dyadic hierarchical linear modeling. Correlational analyses.	Total sample (N)=94, (47 heterosexual married couples), mean age for wives 43 ± 6 years, and for husbands 45 ± 7 years, couples had lived together for an average of 15 ± 7 years.	Significant positive associations were found between husbands’ and wives’ cortisol levels across the day. Higher cortisol levels in husbands were noted when they reported feeling lonelier. Higher cortisol levels in wives were associated with moments spent alone. Cortisol synchrony was stronger among husbands who spent more time with their spouses.
Engert et al., 2014 (Germany) (58)	To investigate the existence of empathic stress, and to determine whether this response is influenced by emotional closeness between the observer and the target, the modality of observation (real-life vs. virtual), and the observer’s sex.	Multicenter design (two research institutes). One member of the couple or a stranger underwent a stress-inducing task (Trier Social Stress Test – TSST) while the other watched it (real-life or virtual). Salivary samples for cortisol and amylase collected before and after the interaction tasks, at multiple time points. Statistics: Linear mixed effects	Total sample (N)=422, 50% female, age 18–35 years. High closeness task (111 opposite-sex couples, N=222), low closeness task (100 opposite-sex strangers, N=200) Age between 18 and 35 years.	54 out of 211 observers (26%) exhibited a physiologically significant increase in cortisol after watching a target undergo the Trier Social Stress Test (TSST). Increase in cortisol was more pronounced in observers with emotional closeness to the targets and in real-life observation.
Schneiderman et al., 2014 (Israel) (59)	To explore the mutual influences between partners’ hormonal levels (oxytocin, prolactin, testosterone, cortisol, and dehydroepiandrosterone sulfate) and how these hormonal dynamics contribute to conflict behavior and the duration of romantic relationships during the initial stages of love.	Couples participated in a conflict interaction task where they were prompted to select an area of disagreement and discuss it in a naturalistic manner for 7 minutes. The aim was to observe their conflict behavior, which was later coded for levels of empathy and hostility. Blood samples were collected to assess plasma levels of oxytocin, prolactin, testosterone, cortisol, and dehydroepiandrosterone sulfate (DHEAS). Follow-up: six months after the initial assessment, a second blood sample was collected from (25) couples who remained together. Statistics: Actor-Partner Interdependence Model. ANOVAs. Correlational analyses.	Total sample (N)=240 (120 heterosexual couples, in early stages of relationships), mean age 24.1 ± 6.7 years, average duration of the romantic relationships at baseline 2.4 ± 0.7 months.	Higher plasma cortisol levels and DHEAS levels were directly associated with increased hostility during conflict discussions. Conversely, individuals whose partners had elevated oxytocin levels tended to show increased empathy in their conflict interactions. High testosterone in one partner predicted greater hostility only when the partner also had high testosterone levels. Higher combined cortisol levels in both partners was associated with relationship breakups, mediated by a decrease in empathy.
Laws et al., 2015 (USA) (60)	To test the hypothesis that spouses converge in their cortisol responses over time, particularly during conflict discussions, and to explore how relationship factors like cohabitation length and relationship dissatisfaction affect this convergence.	Couples participated in a conflict discussion in a laboratory setting at two points: approximately 6 months after marriage and again about 2 years into marriage. Saliva samples were taken to measure cortisol levels before, during, and after the discussions. Statistics: Dyadic analytic methods.	Total sample (N)=366, 50% female, average age 29.36 years (males) and 27.98 years (females), divided in 183 opposite-sex married couples.	The study found significant convergence in couples’ cortisol trajectories, indicating increased similarity in cortisol reactions during conflict discussions as their relationship matured. Longer cohabitation was associated with stronger convergence in cortisol slopes before the conflict discussion. Couples with greater relationship dissatisfaction exhibited higher levels of convergence in acute cortisol responses during conflict.
Coutinho et al., 2017 (Portugal) (61)	To examine the relationship between physiological arousal and the interactions of couples during a marital task, focusing on how these physiological responses vary between positive and negative interaction phases.	Couples had a structured conversation task about negative and then positive aspects of their relationship. The physiological measurements were recorded continuously during the baseline periods and the active interaction tasks (continuous electrocardiogram and electrodermal activity). Salivary cortisol was assessed baseline, after the negative discussion and after the positive discussion. Statistics: SPSS statistical software, with parametric tests, a two-way mixed ANOVA, posthoc t-tests and Pearson’s correlation test.	Total sample (N)=64, 50% female, average age 32.3 years (males) and 33.3 years (females), divided in 32 opposite-sex couples.	The study found variations in heart rate (HR) and skin conductance level (SCL) during interaction tasks, with higher HR and cortisol levels during negative interactions compared to positive ones.
Doerr et al., 2018 (Germany, Austria & Switzerland) (62)	To investigate how fatigue, self-reported stress, and biological markers of stress co-vary within couples and to explore the influence of daily life couple interactions on these dynamics.	Couples reported their subjective fatigue and stress levels four times daily over five consecutive days. They also provided information on couple interactions. Salivary cortisol and alpha-amylase were collected as biological stress markers during these assessments. Statistics: Dyadic multilevel models.	Total sample (N)=80 (40 heterosexual couples), mean age 28 ± 5 years.	A significant co-variation of fatigue and self-reported stress levels was found within couples. Co-variation was stronger when partners had interacted recently (role of social dynamics). Salivary cortisol levels were found to co-vary between partners, but the regulation of alpha-amylase levels depending on the partner’s levels was significant only in females. Couple interactions of negative quality correlated with higher fatigue levels.
Engert et al., 2018 (Germany) (63)	To investigate whether women who exhibit cortisol stress resonance in laboratory settings also show a greater link to their partner’s diurnal cortisol rhythm in everyday life.	Couples completed the Trier Social Stress Test (TSST) in a laboratory and collected diurnal salivary cortisol samples over two non-consecutive weekdays, assessing stress responses under both real-life and virtual conditions of partner presence. Statistics: Hierarchical Linear Modeling.	Total sample (N)=88, 50% female, average age 25.7 years. Divided in 44 opposite-sex couples.	There was a strong connection between physiological stress responses in controlled environments and daily life hormonal patterns in couples, suggesting that close relationships can influence health outcomes through shared physiological mechanisms.
Phan et al., 2019 (USA) (64)	To investigate physiological attunement between romantic partners during a stress-inducing task and to explore whether factors such as sex, social support, and physical proximity influenced this attunement.	Partnered TSST participants: only one partner from each dyad participated in the Trier Social Stress Test (TSST) while the other partner observed from a separate room. Random assignment was used. Solo TSST participants: a group of individuals that were not in a romantic relationship and who participated alone in the stress test. Saliva samples for cortisol assaying were collected at five-time points. Heart rate (HR) and respiratory sinus arrhythmia (RSA) were continuously monitored throughout the experimental session. In couples, factors such as social support and physical proximity were assessed. Statistics: Mixed effects models.	Partnered TSST: total sample (N)=126, (63 heterosexual couples), mean age 22 ± 3 years Solo TSST: total sample (N)=63, mean age 21.9 ± 2.2 years,	The presence of the romantic partner increased the participant’s cortisol levels compared with individuals who completed the TSST alone. Significant RSA attunement was observed between romantic partners in the experimental session. Lower reported levels of social support were associated with higher cortisol attunement between romantic partners. Regarding HR attunement, there were gender differences. When male participants took part in the TSST and their female partners were present, there was a stronger correlation in their heart rates. That did not happen when females participated in the TSST.
Bierstetel et al., 2020 (USA) (65)	To examine whether the positive and negative behaviors exhibited by couples during laboratory conflict interactions are associated with individuals’ diurnal cortisol patterns in everyday life.	Couples provided 18 salivary cortisol samples over a 3-day period. Then the participants engaged in two ten-minute dyadic conflict discussions that were coded for behaviors. They analyzed the relationship between couples’ behaviors during conflicts and the resulting diurnal cortisol patterns. Salivary cortisol was measured at multiple time points. Statistics: Multilevel modelling	Total sample (N)=164, 50% female, average age 35.1 years (males) and 34.1 years (females), divided in 62 opposite-sex married couples.	Positive conflict behaviors were significantly linked to healthier cortisol patterns, while negative behaviors did not show significant associations. Affection was associated with increased cortisol slopes, whereas scorn was connected to flatter cortisol slopes.
Pauly et al., 2020 (Canada and Germany) (29)	To investigate daily interpersonal physiological dynamics (cortisol synchrony) in older couples, examining its associations with factors such as partner presence, daily positive partner interactions, and individual differences in empathy.	Two-center study. Couples participated in daily assessments where they completed questionnaires and provided salivary cortisol samples five to seven times daily over a period of 7 days. Salivary cortisol was measured at multiple time points. Statistics: Multilevel modelling	Total sample (N)=324, 50% female, average age 71.2 years in one center and 74.4 in the other center, divided in 162 older adult couples, most opposite-sex (1 same-sex couple).	There was significant dyadic covariation in cortisol levels, indicating that cortisol synchrony is present among partners. Partner presence was associated with greater cortisol synchrony only in one of the centers. Higher cortisol synchrony was correlated with prior positive socioemotional partner interactions in both centers. No significant association was found between cortisol synchrony and empathic concern or perspective taking.
Shrout et al., 2020 (USA) (66)	To examine how individuals’ perceived stress and their partners’ perceived stress affect cortisol levels and slopes across the day, as well as how positive and negative behaviors during conflict discussions influence these relationships.	Dyadic study involving both partners of married couples. Participants completed a full day in-person visit, where they engaged in a marital conflict discussion, and their cortisol levels were measured. Salivary samples for cortisol collected before and after the tasks, at multiple time points. Statistics: Multilevel modelling	Total sample (N)=86, 50% female, average age 38.2 years. Divided into 43 married couples.	Individuals with more stressed partners exhibited flatter cortisol slopes across the day, indicating dysregulation. Average cortisol levels were significantly higher at intervals after the conflict discussion in those partnered with more stressed individuals. Negative behavior amplified the effects of a partner’s stress on an individual’s cortisol levels.
Wang et al., 2020 (China) (67)	To investigate how individuals’ insecure parental and peer attachment influences their own and their partners’ physiological stress responses (cortisol recovery) to romantic conflicts, and whether this relationship is mediated by fearful romantic attachment.	Couples participated in two laboratory tasks designed to induce stress related to their romantic relationship. Saliva samples were collected at five different time points during the laboratory session, for cortisol assaying. Couples were assessed on their attachment to parents, peers, and romantic partners. Statistics: Actor–partner interdependence mediation models. Correlational analyses.	Total sample (N)=242, (121 heterosexual couples), mean age for males 22.3 ± 2.4 years and for females 21.6 ± 2.2 years.	Women’s insecure attachment to parents and peers was significantly associated with a blunted cortisol recovery from romantic conflict, mediated by fearful attachment with their romantic partners. Conversely, men’s insecure attachment to parents and peers correlated with their partners’ adaptive cortisol recovery from romantic conflict, mediated by men’s fearful romantic attachment. Physiological responses and attachment styles among young couples vary significantly by gender, with women’s responses being more adversely affected by attachment insecurities.
Pauly et al., 2021a (Canada & Germany) (68)	To examine the associations between everyday cortisol synchrony and the changes in relationship satisfaction and non-high-density lipoprotein (non-HDL) cholesterol levels over a 3-year period in older couples.	The study employed a longitudinal design in which participants were assessed at multiple time points (T1, T2, T3) over three years. The assessment included: daily life assessments for cortisol sampling over 7 days; blood samples for cholesterol level analysis at T1, T2, and T3; self-reported relationship satisfaction evaluations. Statistics: dyadic latent growth curve structural equation modeling.	Total sample (N)=170, (85 heterosexual couples), mean age 71.2 ± 6.1 years.	Among wives, higher cortisol synchrony was linked to significant increases in relationship satisfaction over time. For husbands, cortisol synchrony did not show a significant association with changes in relationship satisfaction. Higher cortisol synchrony in wives was associated with significant increases in non-HDL cholesterol levels. Husbands demonstrated higher non-HDL cholesterol levels at Time 1 related to cortisol synchrony, but this was not associated with changes over time.
Pauly et al., 2021b (Switzerland, Germany, USA, Canada) (69)	To investigate how different political contexts, as represented by the left-right political orientation of federal states in Germany, are associated with cortisol synchrony among older couples.	Micro-longitudinal study utilizing a repeated daily assessment approach. Each individual provided saliva samples for cortisol measurement up to 7 times a day over a period of 7 consecutive days. They also completed bio-psychosocial questionnaires and provided personal information in annual interviews (as part of the German Socio-Economic Panel).	Total sample (N)=320 (160 heterosexual couples), mean age 72.3 ± 5.8 years.	Cortisol levels were significantly linked between partners. Cortisol synchrony was higher in couples residing in federal states politically located further to the right on the left-right spectrum. There was no significant association between individual L-R political orientation and the cortisol synchrony of couples. Context-based factors (political orientation of the environment) had a more pronounced impact on physiologic synchrony than the individual political beliefs of the partners themselves.
Yoneda et al., 2024 (USA, Canada & Germany) (70)	To investigate the daily dynamics of emotional experiences and cortisol secretion among older adult couples, examining how an individual’s emotions relate to their partner’s cortisol levels, to understand within-couple dynamics of these experiences.	Data from three similar though independent studies originating from Canada and Germany was combined. Longitudinal design with repeated assessments over seven consecutive days. Reports were collected through short electronic surveys that participants completed up to five times daily. Salivary cortisol was measured at multiple time points. Statistics: Three-level multilevel modelling and Actor-partner interdependence models.	Total sample (N)=642, 50% female, average age 72.4 years, divided in 321 older adult opposite-sex couples, most married.	Emotional experiences of one partner significantly influenced the cortisol levels of their partner. Cumulative negative emotions reported by one partner linked to increased overall physiological arousal (elevated cortisol levels) in their partner (potential detrimental impact on partners’ health). Conversely, higher positive emotions in one partner associated with lower cortisol levels in both partners (protective effect against stress).

Across studies, consistent evidence emerged supporting the presence of cortisol attunement between partners, with several investigations reporting co-variation in diurnal cortisol rhythms and acute cortisol reactivity during conflict discussions or stress-inducing tasks (e.g., 28, 56, 66). Attunement tended to increase under emotionally intense or stressful conditions, particularly in couples experiencing high marital strain, low social support, or strong attachment insecurities (e.g., 67, 71). Interestingly, higher cortisol attunement was sometimes linked to maladaptive outcomes—such as elevated inflammation markers or dysregulated HPA patterns—especially when partners were emotionally distressed. Conversely, positive relational behaviors, empathic exchanges, and shared routines were associated with healthier cortisol synchrony and better relational outcomes over time, particularly in older couples (e.g., 29, 68). A recurring pattern across contexts was that partners influence one another’s cortisol levels both in the moment and over time, highlighting the biobehavioral interdependence inherent in close relationships.

### 3.4 Autonomic attunement in non-romantic relationships (friends, strangers, and groups)

Fourteen studies evaluated autonomic attunement in non-romantic social settings, including close friendships, strangers, and groups. **Table 4** briefly describes the 14 included studies. Experimental paradigms ranged from cooperative games and joint tasks to emotionally salient conversations and stress-inducing scenarios. Most studies were conducted in North America and Europe, with participants primarily composed of university students or young adults (mean ages typically in the early to mid-20s). Several studies used same-sex dyads or small teams, few studies included cross-gender pairings.

**Table 4:** Details of the studies examining physiological attunement of autonomic nervous system parameters between friends, strangers and in groups.

Authors, year and country	Purpose of the study	Study design and statistical analysis	Sample characteristics	Major findings
Mønster et al., 2016 (Denmark) (72)	To investigate the coordination dynamics of multiple psychophysiological measures and their utility in capturing emotional dynamics in teams.	Participants were tasked with cooperative production, specifically constructing origami boats in a team of three. In the task, participants were assigned to three specific roles, which were organized in a sequentially interdependent manner, similar to a production line. During the initial instruction phase, positive or negative emotions were induced. Throughout the production periods, physiological measures were recorded (heart rate, skin conductance, and facial muscle activity). Statistics: ANOVA, Pearson correlation, logistic regression.	Total sample (N) = 153, 78 males, 75 females, participants not previously acquainted, average age 24 ± 4.5 years.	Synchrony in physiological measures, specifically skin conductance and zygomaticus EMG (associated with smiling), was significantly higher among members of the same team compared to those from different teams. Smiling synchrony related to positive affect toward the group while skin conductance synchrony related to group tension and negative affect. When a new team routine was adopted, a decrease in synchrony was seen.
Guastello et al., 2018 (USA) (73)	To explore the connections between autonomic synchronization within teams, team participation levels, and team performance during emergency response simulations.	Participants were organized into 11 teams, each containing 3 or 4 members. They played against an opponent in six simulated emergency response tasks. Group performance was assessed. Electrodermal activity data was recorded throughout the experiment. Statistics: ANOVAs.	Total sample (N) = 55 (6 groups of four members, 5 groups of three members), participants not previously acquainted, mean age 20.4 ± 1.9 years, 53% female.	Greater physiological synchronization among team members was associated with better team performance and higher participation levels. Groups of four members exhibited higher synchronization compared to groups of three. Physiological synchronization observed not only within the teams themselves but also between team members and their opponent (especially in tougher challenges).
Scarpa et al., 2018 (USA) (74)	To investigate whether physiological linkage (PL) can be observed among strangers during interactions and whether there are gender differences in this phenomenon.	The study utilized dynamic linear time series modeling. It focused on assessing cardiac interbeat interval (continuous ECG) linkage in dyads while they engaged in emotional or neutral interactions (speaking or writing about life events). Statistics: dynamic linear regression models.	Total sample (N) = 52 (26 same-gender stranger dyads) (17 female dyads and 9 male dyads), average age 19.5 ± 1.2 years.	The study found statistically significant small PL effects for both male and female dyads. The PL effect was stronger for female dyads (extending to a lag of 4 seconds), while it was weaker for male dyads (extending only to a lag of 1 second). No significant differences were identified based on the type of task or emotional content.
Thorson & West, 2018 (USA) (75)	To explore whether increased physiological linkage to interaction partners negatively impacts the stability of one’s own sympathetic nervous system responses.	Participants engaged in dyadic face-to-face interactions to solve math problems for 30 minutes. A moment-to-moment analysis of pre-ejection period (PEP) was used to assess sympathetic nervous system (SNS) activity. Statistics: Actor-Partner Interdependence Model.	Total sample (N) = 94 mean age 20.1 ± 1.5 years, 68% female. Sample was divided into 47 unacquainted dyads (17 same-gender and 30 cross-gender).	A significant negative correlation between physiological linkage and stability. The more influenced participants were by their partners (higher physiological linkage), the less stable their own physiological responses were.
Vanutelli et al., 2018 (Italy) (76)	To investigate the relationship between physiological linkage (synchrony of autonomic measures) during competitive situations and how this linkage correlates with behavioral activation and the performance of the subjects.	Subjects were randomly divided in dyads and performed a competitive attentional task. Each dyad consisted of two individuals who competed directly against one another in the task. Performance was assessed in terms of accuracy and reaction times. Trial-related feedback was provided to participants. Autonomic activity parameters were measured throughout the experiment (skin conductance level, skin conductance response, heart rate). Behavioral inhibition and activation were assessed through a questionnaire. Statistics: ANOVAs, correlational analysis.	Total sample (N) = 32, average age 21.1 ± 3.2 years, 69% female. Divided into 16 dyads. Participants were not previously acquainted.	Inter-subject analyses revealed significant physiological synchrony (skin conductance response and heart rate) after the feedback was provided. Intra-subject analyses showed improved behavioral performance following the general evaluation assessing a winning condition. Higher Behavioral Activation System (BAS) reward scores correlated with increased electrodermal activity, suggesting a motivational impact of winning on physiological responses.
Danyluck & Page-Gould, 2019 (USA & Canada) (77)	To investigate how social context influences physiological synchrony and to discern whether the patterns of autonomic reactivity can predict social processes such as affiliation during interactions between strangers.	Participants engaged in a knot-tying task under different social contexts while their autonomic nervous system activity was monitored continuously using electrocardiograph (ECG). Participants were randomly assigned to conditions aimed at varying the social interaction dynamics (talking vs. no talking; cooperative vs. competitive). Statistics: multilevel modeling.	Total sample (N) = 134, (67 same-sex, same-ethnicity pairs, not previously acquainted), mean age 20.5 ± 5.7 years, 70% female.	Physiological synchrony was observed across both social contexts with variations in sympathetic and parasympathetic reactivity. Conversation and cooperation elicited the greatest sympathetic reactivity while conversation and competition elicited the greatest parasympathetic reactivity.
Behrens et al., 2020 (Netherlands) (78)	To investigate the relationship between physiological synchrony (in skin conductance and heart rate) and cooperative success during social interactions, assessing whether these dynamics are influenced by visual contact.	Participants engaged in the Prisoner’s Dilemma game in dyadic interactions, with conditions where they could see each other (face-to-face) or were blocked from seeing one another. Heart rate and skin conductance level were measured throughout the task. Statistics: Correlational analysis.	Total sample (N) = 152, (76 same-sex dyads, not previously acquainted), mean age 23 ± 4.3 years, 71% female.	The interaction effect between skin conductance level synchrony and visual conditions significantly predicted cooperative success. Cooperative success was positively associated with levels of synchrony but only when participants could see each other (face-to face interactions), not in the face-blocked conditions. Only skin conductance synchrony (not heart rate synchrony) significantly correlated with cooperative success.
Oveis et al., 2020 (USA) (79)	To investigate how one individual’s emotion regulation strategies (specifically stress reappraisal) affected their teammates’ physiological stress responses in a collaborative context, exploring the interpersonal dynamics of emotion regulation during stress.	Participants worked in same-gender, same-race/ethnicity dyads to complete the following tasks: (a) collaborative work designing a new product, marketing plan, and pitch for the product; and (b) an individual presentation of the product. In the experimental design, one of dyad members (manipulated teammate) was randomly assigned to one of the three following conditions: reappraise their stress, suppress emotional display or a control condition. Physiological parameters (pre-ejection period, cardiac output and total peripheral resistance) were assessed at baseline and during the first minute of the tasks. Statistics: Multilevel linear model, ANOVA, logistic regression.	Total sample (N) = 266, undergraduate students, not previously acquainted, average age 20.9± 3.0 years, 60% female.	Teams where one member engaged in stress reappraisal exhibited more adaptive cardiovascular responses (higher cardiac output, lower total peripheral resistance) compared to teams in the suppression and control conditions. Stress reappraisal demonstrated relational benefits, with both the manipulated (those instructed to reappraise) and nonmanipulated teammates showing improved physiological responses. The physiological changes were noted during both collaborative and subsequent individual performance tasks, indicating lasting effects of interpersonal interactions.
Gordon et al., 2021 (Israel, Germany & Canada) (80)	To investigate how physiological synchrony among group members relates to positive affective behaviors exhibited during group decision-making tasks and to explore the role of individual anxiety traits in moderating these effects.	Participants completed the Desert Survival Task (DST), a task widely used to examine group dynamics. The task involved an Individual Phase and a Group Phase (where they engaged in a group discussion to reach a consensus about an hypothetical extreme situation). Electrocardiography (ECG) and Electrodermal Activity (EDA) were measured throughout the task. Statistics: Multidimensional Recurrence Quantification Analysis (MdRQA).	Total sample (N) = 60 (20 groups of three participants), not previously acquainted, mean age 22.5 ± 2.2 years, 77% female.	Group-level physiological synchrony was a strong predictor of positive affective behavior, while individual and dyadic levels were not significant. Specifically, heart rate synchrony positively predicted positive affective behavior, whereas electrodermal activity synchrony had a contrasting negative effect. Individuals with higher levels of trait anxiety exhibited a greater display of positive affective behaviors (e.g., smiling) when the physiological synchrony of the group was low.
Zerwas et al., 2021 (USA) (81)	To investigate the relationship between physiological attunement and empathic accuracy in female friends during a stressful task. Regarding attunement, the authors made a distinction between physiology – physiology linkage (degree of covariation in physiological responses between two individuals) and physiology – experience linkage (the level of connection between perceivers’ physiological responses and targets’ emotional experiences)	Controlled laboratory design where participants completed two key tasks: a stressful speech task to induce physiological responses; an empathy task where participants rated their own and their friend’s emotional experiences based on video recordings of their speeches. Autonomic activity (inter-beat interval, finger pulse amplitude, skin conductance level, among other methods) was measured during both tasks. Statistics: multilevel models, correlational analyses.	Total sample (N) = 96 (48 pairs of female friends), average age 42 ± 15.4 years, all female.	A significant association was found between physiology-experience linkage and empathic accuracy, suggesting that perceivers’ physiological responses can act as indicators of targets’ emotional experiences. Physiology-experience (but not physiology-physiology) linkage was associated with greater empathic accuracy, even after controlling for potential confounding factors.
Goldring et al., 2022 (USA) (82)	To investigate how shared reality during co-experienced stressors affects stressor reactivity, with a focus on understanding the psychological processes underlying this phenomenon and examining differences between sexes.	Study 1: A psychophysiological experiment in which subjects participated in a stressful speaking task where shared reality was manipulated through social validation. Physiological measures (heart rate reactivity and pre-ejection period reactivity) were recorded to assess stress responses. Study 2: A 14-day dyadic daily diary study with romantic couples from New York City. Couples reported their co-experienced stressors and feelings of shared reality each day during the first year of the COVID-19 pandemic. Statistics: Multivariate multilevel model, Bayesian estimation.	Study 1: Total sample (N) = 70, not previously acquainted, average age 20 ± 2 years, all female. Study 2: Total sample (N) = 204 (102 heterosexual romantic couples), average age 28 ± 8 years.	In Study 1, participants reported feeling less anxious when they experienced shared reality with another person facing the same stressor. Women exhibited reduced heart rate and improved parasympathetic activation when shared reality was present. In Study 2, nearly all females (99% of the sample) reported reduced anxiety in the presence of shared reality, compared to only 42% of males who did so.
Flory et al., 2023 (Switzerland) (83)	To investigate the processes underlying physiological synchrony during a self-paced synchronized finger tapping task and to determine whether interindividual cardiac physiological synchrony resulted from the task-related stimuli shared among individuals.	A self-paced interpersonal tapping synchronization task was applied. Four conditions were tested: bimodal visual-auditory, unimodal auditory, unimodal visual, and a control condition with no sensory communication. Heart rate variability parameters were tested. The degree of physiological synchrony observed in original pairings (i.e., initially selected dyads) was compared to that observed in randomly combined dyads. Statistics: Wavelet transform coherence analysis. Kruskal-Wallis test, Wilcoxon test.	Total sample (N) = 40 (20 dyads) (19 men, 21 women), average age 23.7 years (range 18-35). The study report does not specify whether the participants in each dyad were friends or had any prior social relationship.	Significant increases in heart rate synchronization from baseline to task execution were observed. No statistically significant difference across the various conditions was seen regarding synchrony levels. Synchrony between randomly combined dyads was similar to that of the original dyads, indicating that the observed cardiac synchrony might be a universal phenomenon arising from the task-dependent mechanism.
DiGiovanni et al., 2024 (USA) (84)	Experiment 1: To explore the physiological synchrony between individuals during supportive conversations. Experiment 2: To explore the differences in physiological responses between close friends and romantic partners when discussing problems, particularly focusing on the influence of co-rumination.	Both experiments emphasized a comparative analysis of physiological synchrony in different types of relationships (friends vs. romantic partners), utilizing similar methodological approaches. Key features included: random assignment to experimentally controlled conditions (co-rumination vs. natural); use of physiological measures (pre-ejection period reactivity) to assess emotional and sympathetic arousal during structured conversations. Statistics: Mixed-effects models, multilevel modeling, correlational analyses.	Experiment 1: Total sample (N)=294 (147 close friend dyads), average age 18.9 ± 1.4 years, 78.9% female. Experiment 2: Total sample (N)=226 (113 romantic dyads), average age 20.1 ± 2.4 years, 88.5% heterosexual dyads.	Experiment 1: Close friend dyads exhibited significant physiological covariation in pre-ejection period (PEP) reactivity when engaging in supportive conversations. The co-rumination condition did not cause more synchrony between dyads members. Experiment 2: Romantic partner dyads also demonstrated significant physiological covariation in PEP reactivity during their supportive conversations. Romantic partner dyads exhibited significantly less positive physiological covariation (i.e., less reciprocate response) as compared to close friends.
Boukarras et al., 2025 (Italy) (85)	To investigate how different contextual demands during joint actions (like task novelty and roles in interaction) and individual differences (like Social Anxiety and Perspective Taking) influence physiological synchrony among dyads.	Participants participated in a joint grasping task consisting of 16 blocks (each block containing 20 trials). The task was designed to evaluate how participants synchronized during conditions that varied with respect to roles in the interaction, movement types, task novelty. Heart rate variability was measured through continuous ECG data. Statistics: Mixed-effects linear models.	Total sample (N) = 80 (40 same-sex, unacquainted dyads) (17 male-male and 23 female-female), average age 25.8 ± 4.2 years.	Significant increases in cardiac synchrony were noted during task novelty compared to repeated tasks. Higher perspective taking abilities were associated with increased HRV during novel task blocks (potentially facilitating better synchronization with their partner’s physiological states). Higher levels of social anxiety were associated with a decrease in physiological synchrony. Synchrony was higher in interactions with a peer-to-peer than in those with a leader-follower dynamic.

A consistent finding across studies was the emergence of autonomic attunement during shared emotional or interactive experiences. For instance, greater physiological synchrony was associated with enhanced team performance (73), successful cooperation (78), or positive affective behavior during group deliberation (80). However, attunement was not uniformly beneficial—one study suggested that higher physiological attunement could undermine autonomic stability (75).

Key modulators of attunement included visual contact, task novelty, social roles, and emotion regulation strategies. Visual access amplified attunement effects (78), and emotion regulation through stress reappraisal led to more adaptive cardiovascular responses at both individual and dyadic levels (79). In group settings, synchrony at the collective level (not just between pairs) predicted affective outcomes, and task-induced synchrony emerged even among stranger dyads (83).

### 3.5 Cortisol attunement in friends and groups

Seven studies investigated the attunement of endocrine responses—particularly cortisol—within friendships, strangers, and groups. **Table 5** briefly describes the 7 included studies. All studies employed salivary biomarkers and used experimental or semi-naturalistic tasks to induce emotional engagement or stress, including structured conversations, co-rumination, psychosocial stress tests (e.g., TSST), and outdoor group activities. Most studies were conducted in the United States, with a predominance of female participants and young adult samples (mean age range: 18–24 years), except for Denk et al. (90), which included a broader age distribution. Methodologically, multilevel modeling, actor–partner interdependence models, and linear regressions were commonly used to analyze dyadic and group-level hormone data.

**Table 5:** Details of the studies examining physiological attunement of cortisol between friends and in groups.

Authors, year and country	Purpose of the study	Study design and statistical analysis	Sample characteristics	Major findings
Ketay et al., 2017 (USA) (86)	To investigate the associations between testosterone and cortisol levels with feelings of closeness and the desire for closeness during friendship formation.	Participants completed either a high closeness (self-disclosure activity) or a low closeness (minimal self-disclosure) task. Salivary samples for testosterone and cortisol collected before and after the interaction tasks. Statistics: Multilevel Linear Modeling.	Total sample (N)=116, 79.3% female, average age 19.7 ± 1.9 years not previously acquainted. High closeness task (26 dyads, N = 52), low closeness task (32 Dyads, N = 64).	Individuals with lower testosterone felt closer and desired more closeness. Lower basal cortisol levels and decreases in cortisol were also linked to greater closeness and desired closeness in interactions.
Anderson et al., 2018 (USA) (87)	To investigate the informative and evocative properties of spontaneous fear vocalizations within a challenging and socially interactive outdoor context (white-water rafting), linking emotional expression with physiological arousal and subjective experience.	The study examines spontaneous emotional expressions in naturalistic settings, specifically during white-water rafting experiences. Participants were recorded while rafting in groups (range of 5 to 9 individuals per raft), allowing for analysis of vocalizations, face, and body expressions related to fear, amusement, pride, and awe. Data collection included video recordings of participants, subjective emotion measures, and physiological assessments (cortisol) pre- and post-rafting. Statistics: ANOVA, Multilevel modeling.	Total sample (N) = 69, consisting of adolescent youth from underserved communities and military veterans. Individuals ‘age was not assessed. Forty nine % female.	Fear vocalizations cohered with facial expressions of fear and subjective reports of fear. After controlling for pre-trip cortisol levels, the number of fear vocalizations was positively associated with cortisol levels measured after rafting. Fear vocalizations clustered within group members in rafts, showing significant between-raft variance in vocal expressions (suggesting contagion). Cortisol levels of participants converged at the end of the trip, indicating a collective physiological response among group members.
Rankin et al., 2018 (USA) (88)	To determine whether adrenocortical attunement occurs in women’s close friendships and to explore the role of co-rumination	Dyads were randomly assigned to a Problem-Discussion condition or a Control condition. Three points of salivary cortisol assessment: pre-task, immediately after and 20 minutes after (follow-up) the tasks. Statistics: bivariate correlations and multiple regression models.	Total sample (N)=74, all female, average age 19.2 years (range 18 to 24). Divided into 37 friendship dyads.	Friends exhibited adrenocortical attunement before and after tasks. Pre-task attunement predicted co-rumination, and co-rumination significantly predicted post-task and follow-up attunement.
Cook, 2020 (USA) (89)	To assess affective and physiological synchrony in late adolescent friendship dyads during a structured stress-inducing task.	Dyad engaged in a structured 15-minute discussion about conflicts within their friendship. Baseline and post-task salivary cortisol and alpha amylase (sAA), as well as self-reported positive and negative affect were measured. Statistics: Hierarchical Linear Modeling.	Total sample (N)=100, 70% female, average age 18.8 ± 0,8 years. Divided in 50 same gender friendship dyads. Average length of friendship: 4 ± 4.8 years.	Friends demonstrated synchronization in negative and positive affect, salivary cortisol and alpha amylase (sAA) responses during the induced stress situation. Lower quality (less supportive, conflicted) friendships were associated with stronger synchrony of sAA and cortisol responses.
Denk et al., 2021 (Germany) (90)	To investigate the influence of acute stress on physiological synchrony between group members, particularly focusing on cortisol and alpha-amylase responses.	Participants were randomly assigned to either a stress condition (Trier Social Stress Test – G) or a control nonstressful condition. During the tasks, visual barriers were employed to prevent direct visual contact among group members. Salivary cortisol and alpha-amylase levels were assessed at multiple timepoints. Statistics: actor-partner interdependence model.	Total sample (N)=138, average age 23.5 ± 4.0 years, 47.1% female. Divided into two experimental conditions: • Stress-inducing task: N=75 • Non-stressful tasks (control condition): N=63 Participants were tested in groups with circa 3.1 participants per group (range 2 to 4).	Significant cortisol and alpha-amylase synchrony was found among participants in the same group. Notably, cortisol synchrony was stronger in the non-stressful control condition.
Rankin et al., 2021 (USA) (91)	To investigate the role of hormonal attunement (cortisol and progesterone) within female friendships in coping with psychosocial stressors, also aiming to assess the role of verbal interaction in the transfer of stress response	One friend underwent a stress-inducing task (Trier Social Stress Test – TSST) while the other participated in a non-stressor task. After that, the friends were reunited in the same room but instructed not to communicate verbally for 20 minutes. Salivary cortisol and progesterone was measured at multiple points (baseline, post-stress task, and during recovery). Statistics: Actor-Partner Interdependence Models.	Total sample (N)=40, all female, average age 20.6 years (range 18-24), divided in 20 close friendship dyads.	Close female friends showed aligned cortisol responses (attunement). For progesterone, a negative (inverse) association was observed. However, without verbal interaction, no direct transfer of stress (stress contagion effect) was noted. Self-reported friendship satisfaction did not correlate with physiological attunement of cortisol or progesterone.
Thorson et al., 2021 (USA) (92)	To investigate adrenocortical attunement between new acquaintances during early relationship formation and to examine whether self-disclosure affects physiological synchrony.	The dyads of participants met each other for the first time in the study. Each dyad was randomly assigned to a high self-disclosure or a low self-disclosure conversation (duration 45 minutes). Salivary cortisol was measured at baseline and 20 minutes after the end of the conversation. Statistics: linear regressions.	Total sample (N)=140, average age 22.9 ± 6.3 years, 55% female. Participants were paired into 70 dyads who met for the first time.	Dyads assigned to high self-disclosure questions reported greater personal information sharing compared to those assigned low self-disclosure questions. Greater self-disclosure was associated with more similar cortisol changes between dyad members following the interaction.

Consistently, cortisol attunement emerged across various relational contexts. Female friendship dyads demonstrated aligned cortisol responses at baseline and following stress exposure, especially when co-rumination was present (88). On the other hand, Cook (89) found that alpha-amylase synchrony was stronger in low-quality friendships, possibly reflecting heightened vigilance or conflict sensitivity. Also, greater affective sharing or self-disclosure was associated with more similar cortisol reactivity between unfamiliar dyad partners (92). Notably, the presence of stress did not always enhance attunement—Denk et al. (90) found stronger cortisol and alpha-amylase synchrony in non-stressful group conditions than in stress-inducing ones, suggesting that synchrony may also emerge in emotionally regulated or low-threat contexts.

Additional important themes emerged from our review of physiological attunement and will be addressed in the following section.

## 4 Discussion

Several implications have emerged inductively from the body of literature reviewed in this study. In the following subsections, we explore key themes that contribute to a more nuanced understanding of physiological attunement in adult relationships. First, we examine how physiological attunement varies according to the emotional valence of interpersonal interactions. Next, we consider the role of individual differences, such as empathy and personality traits, in shaping interpersonal synchrony. We also discuss how the duration and modalities of interaction—such as verbal communication, eye contact, and touch—may influence the emergence of physiological attunement. Additionally, we address the associations between physiological attunement and health outcomes across various relational contexts, followed by a closer look at how physiological attunement may reflect relationship quality in romantic dyads. We then consider the link between group-level synchrony and collaborative performance, and finally, we reflect on sex differences in the propensity to exhibit physiological attunement. Together, these thematic areas offer important insights into the dynamic and context-sensitive nature of physiological attunement and its relevance for mental and relational well-being.

### 4.1 Variation in physiological attunement according to the emotional valence of interpersonal interactions

The evaluation of included studies indicates that autonomic attunement is often stronger during conflictual or emotionally charged negative interactions. For example, during marital conflicts, couples showed higher HRV attunement, and this was associated with elevated inflammatory markers and negative affect, suggesting a stress-reactive form of attunement (30, 37). Electrodermal synchrony also increased during negative conversations in couples, possibly reflecting heightened arousal or vigilance (39). Cortisol attunement was sometimes enhanced during conflict or strain, suggesting that couples may “tune in” physiologically in ways that amplify shared stress (60, 66).

In contrast, positive interactions (e.g., affectionate conversations, support exchanges) tend to promote in-phase synchrony that is associated with positive emotional states and relational benefits. Shared positive emotions were associated with greater physiological attunement, higher-quality marital interactions, and greater satisfaction (41). During positive conversations, heart rate synchrony was linked to higher relationship satisfaction (42). During sexual or intimate activities, higher HRV attunement was associated with greater sexual satisfaction (16, 51). Similarly, cortisol attunement emerged more robustly in couples with higher emotional support, positive daily interactions, or shared routines, often predicting better long-term wellbeing and relational satisfaction (29, 68, 70).

Hence, physiological attunement can either reflect harmony or shared distress, depending on the emotional valence and quality of the interaction. Synchrony is not inherently positive or negative—it is shaped by context and relational dynamics (28, 66, 67). This dual nature has direct implications for flourishing. When interpersonal attunement emerges within a supportive and emotionally safe environment, it may act as a physiological mechanism that reinforces connection and emotional regulation—key ingredients of flourishing. Conversely, in relationships marked by chronic strain or emotional dysregulation, attunement may amplify stress and relational dissatisfaction, potentially undermining well-being. Thus, physiological attunement could be considered a dynamic biological substrate through which relationships shape, sustain, or impair individual flourishing (28, 66, 67).

### 4.2 Influence of empathy and personality traits on interpersonal physiological attunement

Empathy may enhance interpersonal attunement by increasing emotional sensitivity and responsiveness, thus facilitating biobehavioral alignment at both cognitive and physiological levels. Empathic concern, measured by self-report tools such as the Interpersonal Reactivity Index (IRI), was positively associated with cardiac attunement during romantic interactions. When partners demonstrated greater trait empathy, their heart rate (HR) patterns were more likely to align (42). In a study examining couples under pain conditions, dyads with higher trait empathy showed stronger HR and respiratory synchrony, especially when touch was involved (49). Perspective-taking, considered one of the components of empathy (93), was shown to enhance autonomic attunement in couples during conflict discussions. Those induced to take their partner’s perspective exhibited greater alpha-amylase synchrony—a marker of sympathetic activity (30).

During shared empathy-for-suffering tasks, couples showed attunement in electrodermal and cardiac responses (53). Empathic accuracy refers to the ability to accurately infer the specific thoughts and feelings of another person in real time during social interaction (94). In female friendship dyads, empathic accuracy was significantly associated with physiology–experience linkage, suggesting that individuals with greater empathic sensitivity may physically mirror their partner’s emotional experiences (81).

Some studies also identify other traits that significantly influence the depth and quality of physiological attunement. Attachment style plays a critical role. In adults, attachment styles are commonly described as secure, anxious-preoccupied, dismissive-avoidant, or fearful-avoidant (95, 96), reflecting differing degrees of comfort with intimacy, dependence, and emotional expression. Secure adults typically engage in trusting and balanced relationships; anxious-preoccupied individuals seek closeness but often worry about rejection; dismissive-avoidant individuals emphasize independence and downplay emotional needs; and fearful-avoidant individuals desire connection but fear vulnerability, often due to unresolved relational trauma. Avoidant partners demonstrated weaker synchrony during resting and affectionate conditions, while anxious attachment was associated with heightened cortisol reactivity, particularly during emotionally threatening interactions (15, 55, 67). Furthermore, social anxiety was inversely associated with physiological attunement. Dyads with members scoring high in social anxiety exhibited less cardiac attunement, especially during novel or demanding social tasks (85).

The evidence suggests that empathy and certain personality traits may serve as dispositional catalysts or barriers to physiological attunement, with direct implications for human flourishing. Traits such as empathic concern and perspective-taking appear to promote synchrony in autonomic and endocrine systems, enabling individuals to emotionally and physiologically “tune in” to others in meaningful ways (30, 42, 49). This biobehavioral alignment likely fosters social bonding, emotional safety, and mutual regulation, which are key elements of flourishing as a relational and psychological construct (4, 5). Conversely, traits associated with emotional withdrawal or threat sensitivity, such as avoidant attachment and social anxiety, tend to dampen synchrony (15, 55, 85). These findings suggest that flourishing is not only a product of environmental or relational conditions but is also partly shaped by the individual’s internal dispositional landscape. When personality traits support emotional openness and attunement, physiological systems may align in ways that reinforce well-being. In contrast, dispositional barriers to connection may impair this alignment, potentially limiting opportunities for relational growth and psychological thriving (67, 81).

### 4.3 Duration and modalities of interaction as determinants of physiological attunement

Many of the reviewed studies have showed that strangers can develop physiological synchrony within minutes, suggesting that a long history of relational bonding is not a prerequisite. Stranger dyads achieved significant cardiac interbeat interval linkage during brief emotionally themed conversations or writing tasks, even when they were completely unacquainted before the experiment (74). In joint tasks like cooperative or competitive games, strangers displayed skin conductance and heart rate synchrony within a single session (76). In group contexts, first-time collaborators displayed converging cortisol responses and sympathetic arousal after completing problem-solving or adventure-based activities (80, 87). Thus, synchrony can emerge rapidly when interpersonal engagement is high and the context is emotionally salient.

Verbal communication, especially when emotionally meaningful or affiliative, promotes and amplifies physiological synchrony. Couples engaged in verbal conflict resolution or emotional disclosure showed autonomic and hormonal attunement, including synchrony in HR, EDA, cortisol, and alpha-amylase (40, 41, 71). Emotionally supportive conversations among friends enhanced synchrony in cortisol and alpha-amylase (88, 89). In task-based stranger dyads, talking during interaction increased the physiologic attunement corresponding to the sympathetic branch and improved social outcomes, compared to no-talking conditions (77).

Visual contact also exert a powerful role to promote physiological attunement. Gazing into each other’s eyes enhanced HR and respiratory synchrony among romantic partners—even in the absence of speech (15). In cooperative games like the Prisoner’s Dilemma, face-to-face conditions produced significantly stronger skin conductance synchrony and higher rates of cooperation than visual-blocked conditions (78).

In addition, touch consistently emerges as a robust, nonverbal promoter of physiological synchrony as well. When couples were allowed to hold hands or touch forearms, EDA attunement increased significantly—regardless of emotional valence or task content (47). During pain empathy tasks, touch enhanced synchrony in HR and respiration, particularly among couples high in trait empathy (49). Under stress (e.g., Trier Social Stress Test), partner presence and proximity—especially involving physical closeness—was associated with stronger RSA and cortisol attunement (64).

Evidence indicates that physiological attunement is a flexible and dynamic phenomenon that does not depend on long-term familiarity. It is highly sensitive to behavioral engagement, and can be modulated by multiple sensory and communicative channels, both verbal and nonverbal. These findings carry important implications for flourishing as a dynamic and interactionally grounded construct. If physiological attunement can arise spontaneously in short-term interactions—when facilitated by emotional resonance, gaze, speech, or touch—it suggests that the capacity to biologically connect is widespread and adaptable, offering potential for well-being even in newly formed or transient social ties. Encouraging environments that foster empathic communication, visual presence, and safe touch may thus enhance both social cohesion and individual well-being at a physiological level.

### 4.4 Associations between physiological attunement and health outcomes across relationship contexts

Regarding romantic couples, autonomic attunement during conflict was sometimes linked to increased inflammatory markers and greater emotional distress. For example, HRV synchrony during marital conflict predicted higher levels of IL-6, a pro-inflammatory cytokine (37). Couples with high synchrony in negative affect and physiological stress during arguments were more likely to report poor relational quality and psychological distress (30). Insecure attachment styles and unresolved trauma led to weaker RSA synchrony and greater cortisol reactivity, which are markers of emotional and physiological dysregulation (44, 67). Thorson and West (75) found a significant negative correlation between physiological synchrony and individual physiological stability; participants who were more influenced by their partners exhibited less stable physiological responses of their own. In a longitudinal study, higher cortisol covariation predicted higher non-HDL cholesterol levels over time, suggesting that physiological attunement may pose health risks (68). On the other hand, some studies showed that couples who exhibited cortisol synchrony during daily life were more likely to report greater relationship satisfaction, emotional closeness, and psychological wellbeing (29, 70).

With respect to friendship, in studies of close friends, co-rumination and emotional sharing that led to cortisol synchrony were associated with higher emotional burden and stress transmission (88, 89). However, supportive conversations among friends increased physiological synchrony and were associated with relational satisfaction and affective alignment, which may offer protective benefits over time (84).

These findings suggest that physiological attunement is not inherently beneficial or harmful; rather, its impact depends on interaction quality, emotional tone, and individual differences such as attachment and empathic capacity. In this sense, synchronization can either reinforce co-regulation processes that support flourishing or co-dysregulation dynamics that exacerbate stress and health risk.

### 4.5 Links between physiological attunement and relationship quality in romantic dyads

Heart rate and HRV synchrony during positive conversations, mutual gaze, or emotional sharing were linked with greater empathy, relationship satisfaction, and emotional intimacy (15, 42). During sexual activity, HRV synchrony was positively correlated with sexual satisfaction and partner responsiveness, reinforcing its role in emotional and physical intimacy (51). Cortisol attunement across days was associated with daily connectedness and higher relationship satisfaction (29, 70).

Conversely, during conflict interactions, high autonomic synchrony sometimes predicted emotional strain and hostile affect (30, 37). In distressed couples, synchrony in negative affect was related to lower communication quality and greater emotional vulnerability (40).

Importantly, this context-sensitivity has direct implications for flourishing, understood as a state of optimal emotional, relational, and psychological functioning (4, 5). When physiological attunement emerges in healthy, emotionally attuned romantic relationships, it may act as a biological substrate that supports individual and relational flourishing. Conversely, in strained or dysregulated relationships, synchrony may contribute to relational stress and compromise long-term well-being.

### 4.6 Group-level physiological attunement and its relation to task performance

In a study by Guastello et al. (73), triads working on a creative group task showed that HR synchrony predicted higher group output, better collaboration, and greater consensus. Gordon et al. (80) found that in triads completing cooperative problem-solving tasks, higher within-group HR synchrony predicted greater engagement, positive affective behavior, and successful cooperation. Interestingly, group-level synchrony (across all three members) was a better predictor of outcomes than dyadic synchrony. In Behrens et al. (78), participants playing a social dilemma game in pairs showed greater skin conductance synchrony during face-to-face interactions, which correlated with higher cooperation rates and mutual trust.

Also, in collaborative attentional tasks, Vanutelli et al. (76) showed that attunement was stronger during novel tasks and increased when participants received a positive feedback, which related to better adaptation and group cohesion. In consequence, greater physiological attunement in groups is associated with improved performance in collaborative tasks.

These findings converge to indicate that physiological synchrony in groups serves as a marker of shared cognitive and emotional engagement, facilitating smoother interpersonal coordination and more effective collective action. In broader terms, such alignment may support dimensions of flourishing in group and community settings, by fostering prosocial behavior, shared purpose, and mutual responsiveness.

### 4.7 Sex differences in the propensity for physiological attunement

In romantic couples, females were often found to be more physiologically reactive or attuned during emotionally intense interactions. In emotionally evocative conversations, female partners tended to show stronger electrodermal and HRV synchrony, whether they were in a disclosing or a listening role (53). Trait empathy, which tends to be higher in women, was linked to stronger physiological attunement (42), and women’s empathic concern correlated more consistently with in-phase physiological attunement. In conflictual or stressful contexts, men were sometimes less physiologically reactive or showed delayed synchronization compared to female partners (30, 40). However, some findings suggest that men’s physiological states are more influenced by their female partners’ responses (37). In group and cooperative tasks, no consistent sex differences were observed in synchrony levels, though role (leader vs. follower) and task type seemed to influence who synchronized with whom (80, 85).

These sex-related patterns in attunement likely reflect a complex interplay of biological sensitivity and socialized emotional roles. From a flourishing perspective, they underscore the relevance of interpersonal sensitivity and responsiveness as foundational elements of well-being. If flourishing encompasses emotional resonance, relational quality, and mutual regulation (4, 5), then sex differences in physiological attunement may influence how individuals engage in supportive, co-regulatory interactions that sustain psychological health. Understanding these variations may help tailor relational interventions and emotional education strategies that respect individual differences while enhancing pathways toward relational flourishing. Moreover, this is remarkable that the current literature on physiological attunement is largely based on heterosexual dyads, reporting biological sex (male/female), often without attending to gender identity and gender roles.

### 4.8 Lessons learned from physiological attunement and its importance for human flourishing

The findings of this review suggest physiological attunement may be a key biobehavioral pathway linking relationships to flourishing. Across multiple studies, physiological attunement in romantic couples, friends, and social groups was associated with emotional closeness, mutual regulation, empathy, and stress buffering—core elements of meaningful and flourishing relationships (29, 42, 49). In contexts of support, intimacy, and cooperation, attunement appeared to facilitate affective alignment and promote psychological and even physiological health (51, 70).

**Figure 2:**
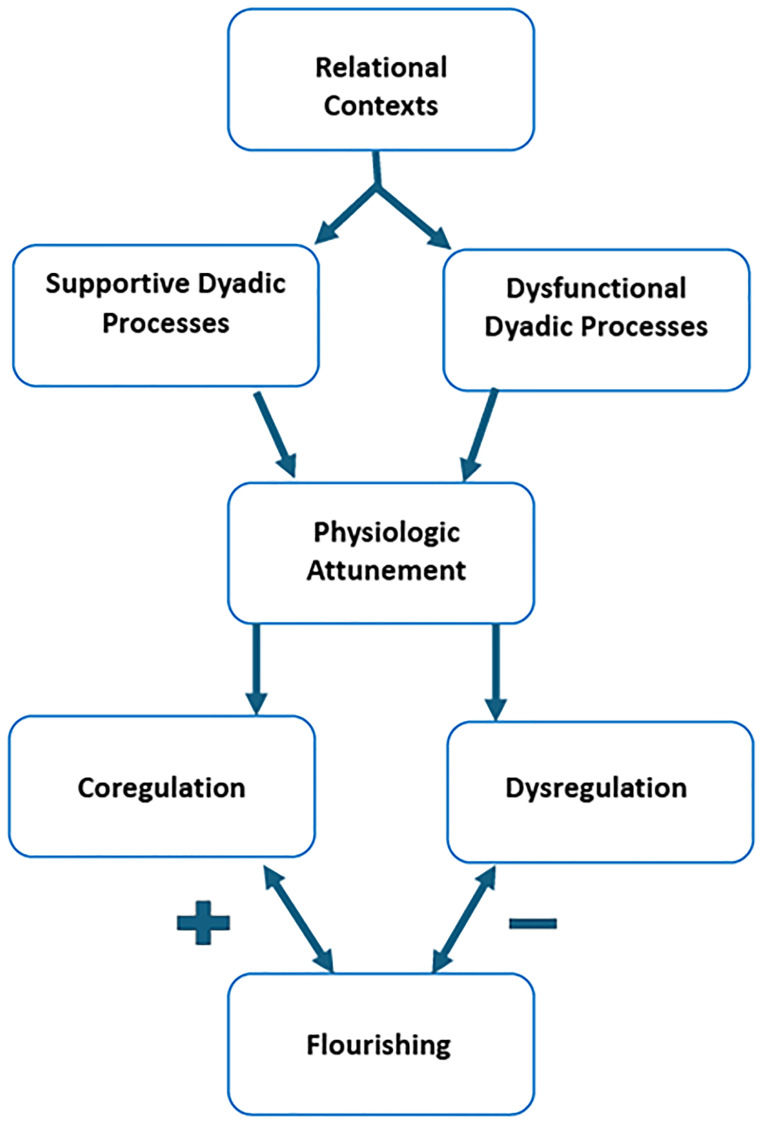
Conceptual model: physiological attunement as a pathway to flourishing.

Conversely, in contexts marked by conflict, unresolved trauma, or insecure attachment, synchrony could reflect co-dysregulation and shared distress (37, 67), which may compromise wellbeing over time. These dual possibilities highlight physiological attunement as a dynamic and context-sensitive process—capable of enhancing flourishing when grounded in emotional safety and reciprocity, but potentially detrimental when reinforcing relational strain. Thus, physiological attunement may not only reflect—but actively support or hinder—the developmental trajectory of individual and relational flourishing. In **Figure 2**, we propose a visual expression of a conceptual model of the relationship between biological attunement and flourishing.

The widespread presence of physiological synchrony across diverse relationship types suggests it may serve an evolutionarily adaptive function in social mammals. Evidence from this review indicates that synchrony emerges even in brief interactions and among strangers (85, 87), supporting the idea of a biologically embedded drive toward alignment that promotes social cohesion, cooperation, and collective regulation. This view aligns with theories in evolutionary psychology and social neuroscience proposing that alignment at behavioral, neural, and physiological levels facilitates trust and coordinated action (24).

*Polyvagal theory* (97) proposes that the mammalian nervous system enables sociality to function as a neuromodulator—calming physiological responses and optimizing bodily functions. According to this theory, social connection allows humans to experience a sense of safety and access positive emotional states that actively suppress threat-related reactions. Social engagement helps shift the autonomic state from defense to one that supports homeostasis. When individuals feel safe, they become more open to others and engage in trusting social interactions that promote coregulation (97). Together with *Social Baseline Theory* (6), these ideas offer a robust conceptual framework for understanding the role of sociality in emotion regulation, well-being, and flourishing.

Although this review focuses on adults, it is worth noting that physiological attunement is likely a lifespan phenomenon, playing distinct roles at different developmental stages. In infancy, synchrony between caregivers and infants supports emotional regulation and attachment formation (24, 98). These early co-regulatory patterns may scaffold future relational attunement. During adolescence, synchrony may become more complex, as individuals negotiate autonomy and social sensitivity (99). In adulthood, it facilitates intimacy, empathy, and pair bonding, with studies showing increased physiological interdependence in long-term couples (42, 51, 68, 70). In older adulthood, this attunement may culminate in deeply embodied mutual regulation, with some evidence suggesting shared physiological decline and increased mortality risk following the loss of a spouse (100, 101). Thus, attunement appears to be a plastic and context-sensitive process, supporting regulation, bonding, and resilience across the human lifespan.

Synchrony increases the predictability of social interaction: It is easier to know what your partner is going to do next if you are synchronized. But this reduction in prediction error comes at the expense of redundancy and reduced information exchange (14, 102, 103). No behavior is optimal in all contexts. What is considered optimal depends on the specific goal or context in which the behavior occurs. If a dyad`s goal is to increase feelings of closeness and similarity, increasing interpersonal synchrony may be optimal. Contrastingly, decreasing interpersonal synchrony may better serve them if their goal is to generate divergent solutions to a complex problem (14). Successful partners dynamically move in and out of synchrony, for instance, to correct moments of misunderstanding, miscoordination, and dysregulation (14).

As a result of the present review, we were able to identify some gaps in this research field. First, there was variation in how physiological attunement was defined and measured, contributing to conceptual heterogeneity. Second, relatively few studies examined long-term health or wellbeing outcomes, leaving open questions about the sustained impact of physiological attunement. Third, most of the included studies categorized participants as male or female based on biological sex, without accounting for gender identity or sexual orientation. This fact limits generalizability to more diverse populations. This is particularly relevant given that gender identity and gender role socialization are known to influence emotional expression, empathic behavior, and interpersonal regulation.

## 5 Limitations

The present work has some limitations. First, most included studies did not assess flourishing directly and the physiological indicators measured not necessarily align with validated flourishing constructs. In other words, the included studies were not selected or categorized based on whether they addressed flourishing explicitly or implicitly. As a result, theoretical links between physiological attunement and flourishing may lack conceptual strength. Second, the scoping review format is appropriate for mapping literature but does not support causal inference. Hence, the present review does not allow us to draw causal or mechanistic conclusions, neither specific practical implications. Third, given the substantial variation in how physiological attunement is defined and measured, it is possible that some relevant articles were inadvertently excluded from the present review.

## 6 Conclusion

Flourishing, as a multidimensional and stable construct of well-being, has predominantly social characteristics rooted in the biological nature of human beings. Given the importance of mental health for overall well-being and the continuum on which mental health operates, physiological attunement may represent a key mechanism through which sociality influences bodily functioning. It can promote well-being when interpersonal relationships are supportive and healthy, but may also contribute to stress contagion and increase vulnerability to illness in the context of abusive or dysregulated relationships.

## Data Availability

The original contributions presented in the study are included in the article/supplementary material. Further inquiries can be directed to the corresponding author.
